# Morpho-functional traits of the coral *Stylophora pistillata* enhance light capture for photosynthesis at mesophotic depths

**DOI:** 10.1038/s42003-022-03829-4

**Published:** 2022-08-24

**Authors:** Netanel Kramer, Jiaao Guan, Shaochen Chen, Daniel Wangpraseurt, Yossi Loya

**Affiliations:** 1 School of Zoology, Faculty of Life Sciences, Tel-Aviv University, Tel Aviv, Israel; 2 Department of Electrical and Computer Engineering, University of California San Diego, San Diego, USA; 3 Department of Nanoengineering, University of California San Diego, San Diego, USA; 4 Scripps Institution of Oceanography, University of California San Diego, San Diego, USA

**Keywords:** Ecophysiology, Biomechanics, Animal physiology, Software

## Abstract

The morphological architecture of photosynthetic corals modulates the light capture and functioning of the coral-algal symbiosis on shallow-water corals. Since corals can thrive on mesophotic reefs under extreme light-limited conditions, we hypothesized that microskeletal coral features enhance light capture under low-light environments. Utilizing micro-computed tomography scanning, we conducted a novel comprehensive three-dimensional (3D) assessment of the small-scale skeleton morphology of the depth-generalist coral *Stylophora pistillata* collected from shallow (4–5 m) and mesophotic (45–50 m) depths. We detected a high phenotypic diversity between depths, resulting in two distinct morphotypes, with calyx diameter, theca height, and corallite marginal spacing contributing to most of the variation between depths. To determine whether such depth-specific morphotypes affect coral light capture and photosynthesis on the corallite scale, we developed 3D simulations of light propagation and photosynthesis. We found that microstructural features of corallites from mesophotic corals provide a greater ability to use solar energy under light-limited conditions; while corals associated with shallow morphotypes avoided excess light through self-shading skeletal architectures. The results from our study suggest that skeleton morphology plays a key role in coral photoadaptation to light-limited environments.

## Abstract (web summary)

Micro-computed tomography scanning and 3D light simulation models reveals distinct morphotypes of the coral species *Stylophora pistillata* depending on depth, and suggest that coral skeletal micromorphology plays a key role in coral photoadaptation.

## Introduction

Biogenic calcification in corals plays a vital role in facilitating reef biodiversity and complexity^1^. Coral calcification comprises the secretion of calcium carbonate crystals in the form of aragonite^2^, producing a great diversity of geometrical structures and fulfilling the multifunctional purposes necessary to maintain reef health^3^. For example, the structural complexity of reef-building corals, on both the reef scale (m-km) and the coral colony scale (cm-m), provides a broad diversity of habitats for reef-associated organisms. Specifically, small and cryptic fishes, which constitute the main proportion of the coral-reef fauna, rely on the corals’ high structural heterogeneity for their survival^4–6^. In addition to genotypic variations, light conditions and water movement are important factors controlling coral architectural growth^7–10^. For some coral species, growth under different environmental conditions can result in changes in their skeletal structure, a phenomenon referred to as “morphological plasticity”^11^. This phenomenon is believed to be beneficial in enabling such coral species to occupy a wider array of abiotic conditions than those with fixed morphologies^10,12^, and is thus thought to promote the ability of corals to withstand different environments^7,13,14^.

In particular, it has long been suggested that phenotypic plasticity in corals is advantageous for maximizing light interception and use across a broad range of depths and/or light regimes^15,16^. Indeed, the relative abundance of different coral morphotypes can often reflect the environmental conditions in which they reside^7,17–20^. For example, the preponderance of plating colonies in mesophotic coral ecosystems (MCEs; characterized predominantly by blue light and 1–20% of surface photosynthetically active radiation (PAR)^21^), has been attributed to the extremely low-light conditions in their surrounding habitat, resulting in their beneficial growth strategy for maximizing incoming light per unit area of coral tissue surface^17^. Corals that are exclusively found in either shallow or mesophotic depths are commonly termed “depth-specialists”^22^. Such corals exhibit permanent morphological modifications acquired through genetic change (i.e., adaptation) that may have evolved to suit local conditions that significantly differ from those of their ancestral origin conditions^23^. In contrast, coral species that occupy a broad depth range are termed “depth-generalists”, and are found overlapping between the shallow and the upper mesophotic zones^22,24^. In essence, a depth-generalist coral species can inhabit light regimes that vary by up to two orders of magnitude of light exposure^25^.

Analogous to patterns in terrestrial plants, variation in light quantity and quality can drive both physiologically and morphologically based strategies for efficient light utilization in corals^16^. In plants, apart from the well-known physiological modifications (e.g., greater quantities of chlorophyll-*a* pigments), leaves in shaded environments are generally thinner and larger as compared to light-adapted leaves^26,27^. Furthermore, the same features can also appear in leaves subjected to the blue spectrum of light^28^. Similarly, depth-generalist corals inhabiting mesophotic environments often exhibit physiological and structural modifications that are hypothesized to aid in the utilization of light capture^29^, thereby enhancing photosynthetic performance and optimizing colony growth under limited optical conditions. Physiologically, deep-reef corals rely on the ability of their photosymbionts to modify their photosynthetic traits, which typically include increasing cell density and an increase in the effective antenna size (i.e., antenna pigments) per photosynthetic unit (PSU) and an increase of PSUs per cell^30–32^. Such traits lead to both higher quantum yields and photosynthetic rates at low irradiances. Furthermore, some depth-generalist corals, such as *Stylophora pistillata*, often harbor a depth-specialist photosymbiont strain that is more advantageous for low light^33^.

In addition, coral morphology can change in response to irradiance levels. In low irradiances, for example, corals increase their area-to-volume ratio by shifting to a flattened or plate-like morphology, which is considered energetically more efficient for the capture of incident light when its availability is low^34,35^. Thus, modular photosynthetic corals can regulate their internal light regime by varying the extent of self-shading surface on the colony scale towards a photosynthetic optimum^8,20,34,36^.

Although understanding the mechanisms that optimize light capture by corals has been the focus of many studies, as far back as the early 1980s^37–39^, the functional significance of morphology at mesophotic depths has not been thoroughly explored, hindering a comprehensive understanding of the various species’ photoadaptative capabilities. Previous work on photoadaptation at mesophotic depths has been mainly focused on physiological and biochemical alterations^40^, while most of our understanding of the interaction of coral architecture with light is primarily derived from the whole-colony growth form^12,29,34,41^. Research focusing on the extent to which measurable small-scale morphological traits can be informative regarding the light-harvesting mechanisms employed by scleractinian corals remains insufficient, particularly for corals inhabiting the mesophotic environments. Recently, 3D-imaging analyses obtained via advanced technologies such as micro-computed tomography (µCT) and laser scanning, have enabled accurate and detailed information on the coral skeletal structure from micro to macro-scale^42,43^.

Using high-resolution µCT scanning, we aimed to determine the role of morpho-functional traits in coral light-harvesting. Specifically, we assessed the variations in small-scale skeletal structures of the common depth-generalist coral *Stylophora pistillata* from shallow (4–5 m) and mesophotic (45–50 m) depths of the northern Gulf of Eilat/Aqaba (GoE/A). Using our morphometric measurements, we developed simple 3D Monte Carlo simulations to examine the effect of coral architecture on coral light propagation and photosynthetic performance. Our findings revealed coral structural changes between depths that have functional significance for capturing and using light in mesophotic environments. Our study also provides a simple computational approach that can be applied to study light-harvesting for a range of mesophotic corals. Together, our findings provide a novel understanding of how small-scale morphology-based mechanisms facilitate enhanced light-harvesting in MCEs.

## Results

### Skeletal morphometrics

**Fig. 1:**
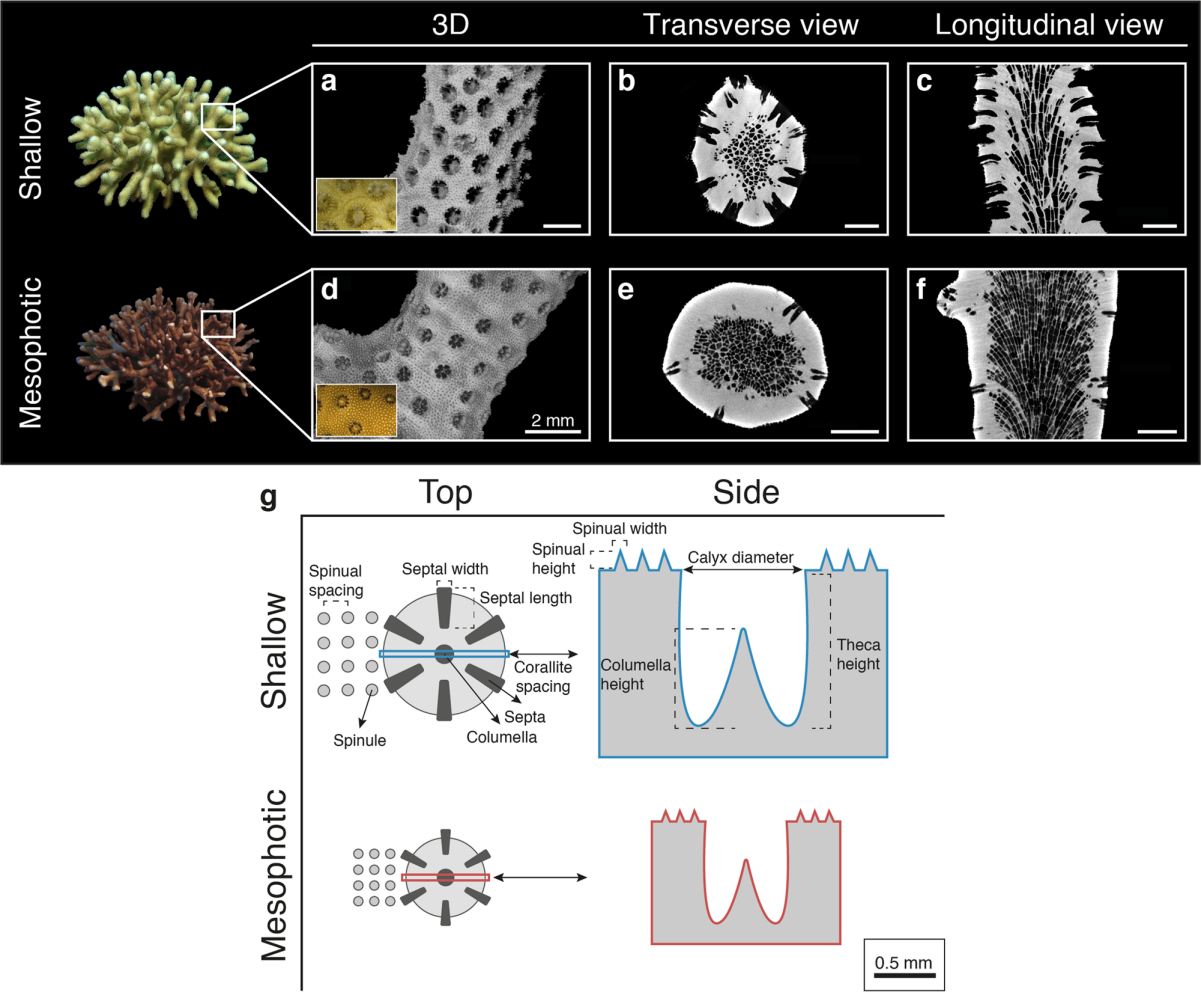
Morphotypes of shallow and mesophotic *S. pistillata*. Examples of μCT X-ray scans from **a**–**c** shallow and **d**–**f** mesophotic showing: **a**, **d** 3D reconstructions of the skeletons (inset photos show surface covered with live tissue) and sections of **b**, **e** transverse and **c**, **f** longitudinal scan slices. Scale bars are 2 mm. **g** Two-dimensional schematic representation of the top-down and side view of a corallite and its surrounding coenosarc between the studied shallow and mesophotic corals. Key skeletal structural elements are noted and scaled based on mean values.

**Fig. 2:**
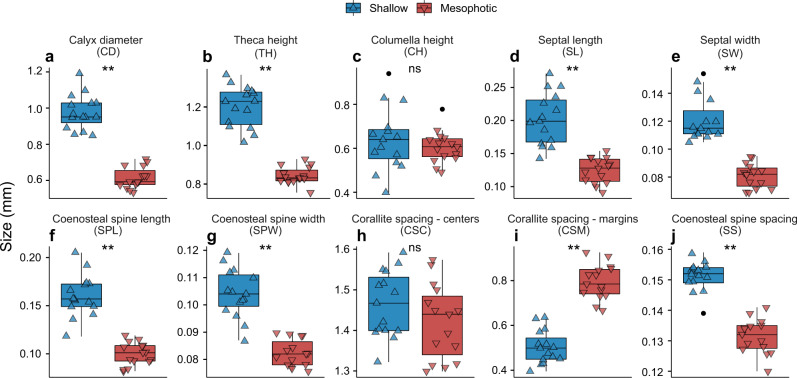
Morphometric results of *S. pistillata*’s skeletal traits. **a**–**j** Box plots showing the mean size variation of morphometric traits between shallow (blue; triangle point up) and mesophotic (red; triangle point down) *S. pistillata* colonies (*n* = 30). Horizontal lines depict the median, box height depicts the interquartile range, whiskers depict ±1.5× interquartile range, and dots represent outliers. Asterisk denotes significance (*p* < 0.01).

**Fig. 3:**
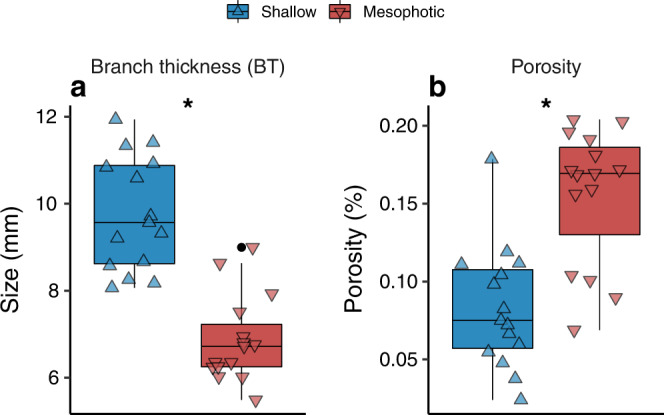
Branch thickness and porosity of shallow and mesophotic *S. pistillata* colonies. Box plots showing **a** branch thickness and **b** porosity between shallow (blue; triangle point up) and mesophotic (red; triangle point down) *Stylophora* corals (*n* = 15 per depth). Horizontal lines depict the median, box height depicts the interquartile range, whiskers depict ±1.5× interquartile range, and dots represent outliers. Asterisk denotes significance (*p* < 0.01).

**Fig. 4:**
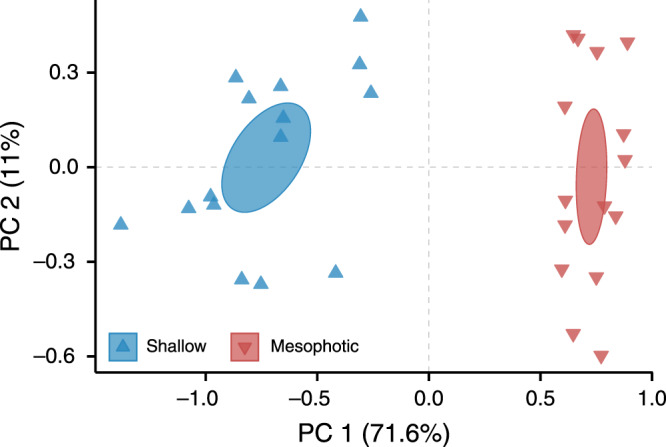
Principal coordinates analysis (PCoA) of the morphological characters of *S. pistillata* based on ﻿Euclidean space. Each color and shape represents a particular colony at a given depth (*n* = 30). Ellipses represent standard error.

Overall, *S. pistillata* colonies exhibited distinct morphotypes between shallow and mesophotic origins, as determined by PERMANOVA (*p* < 0.001; Figs. 1–4). The first two axes of the PCoA captured 82.6% of the total observed variation in the morphological space between shallow and mesophotic colonies. The first axis explained 71.6% of the variance (Fig. 4) and was most correlated with theca height (TH), corallite diameter (CD), and minimal spacing between neighboring corallites (CSM) (contributing 16.1%, 14.2%, and 13.5%, respectively). Similarly, SIMPER analysis identified that most of the differences in small-scale skeleton architecture were attributed to these same traits, which accounted for over a third of the morphological variation observed between depths. Furthermore, while Pearson’s correlation scores were highest and positive between CD, TH, and coenosteum spine length (SPL), they were negatively correlated with CSM (*p* < 0.01; Fig. S1). Excluding CH and spacing between neighboring corallites centers (CSC), all morphometric characters significantly differed between shallow and mesophotic specimens (MEPA, *p* < 0.01; Fig. 2). In general, most of the shallow morphological traits exhibited larger sizes compared to their mesophotic counterparts (Fig. 2). For example, CD was on average ~60% larger in shallow samples, ranging from a diameter of 0.848 to 1.191 mm compared to 0.533 to 0.719 mm in mesophotic samples (Fig. 2a). In contrast, CSM was greater in mesophotic specimens compared to in shallow ones, exhibiting 58% more spaced corallites (Fig. 2i).

Branch thickness was ~30% thinner in mesophotic colonies than in shallow ones (MEPA, *p* < 0.01; Fig. 3a). Lastly, porosity analyses of mesophotic specimens revealed a 7.3% more porous skeleton than in shallow specimens, presenting 8.28 ± 0.01% and 15.57 ± 0.01% (mean ± SE), respectively (MEPA, *p* < 0.01; Fig. 3b).

### 3D models of light capture and photosynthesis

**Fig. 5:**
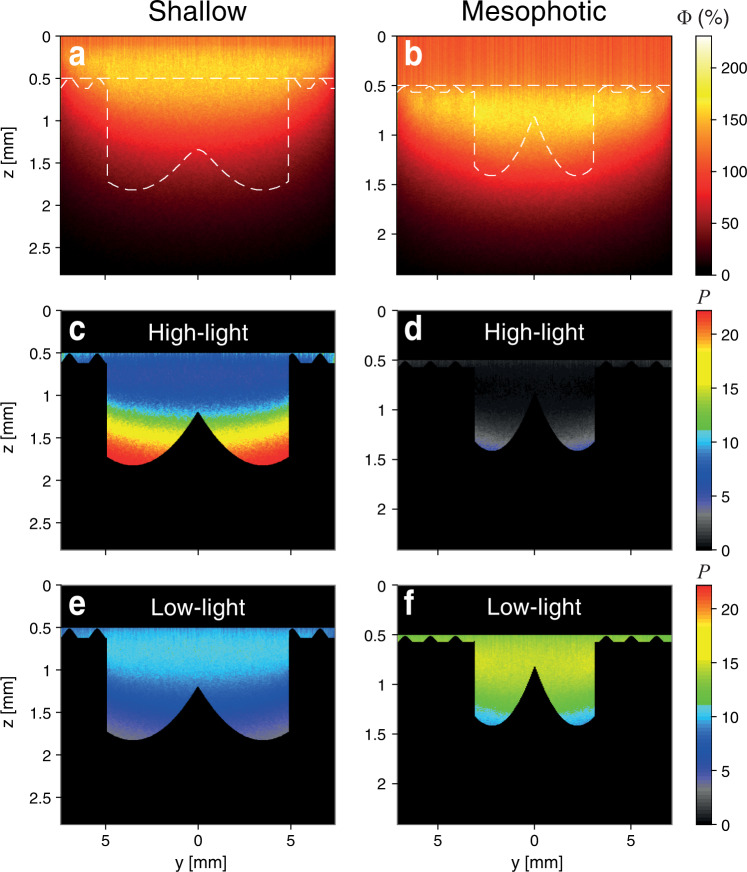
Examples of light propagation simulations shown in 2D (*y*-z axes). Examples of light propagation simulations shown in 2D (*y*-*z* axes) for simplicity (see scores normalized per tissue pixel for all 112 scenarios in Fig. 6). **a**, **b** Relative fluence rates (Φ; delivered as W m^−2^; as “fire” color gradient) with contour indicating the surface boundaries of shallow and mesophotic natural morphotypes. **c**–**f** Photosynthetic score (*P*; as “rainbow” color gradient) on the tissue layer of shallow and mesophotic architectures with default settings under light intensities of 750 (high-light) and 45 (low-light) µmol photons m^−2^ s^−1^.

**Fig. 6:**
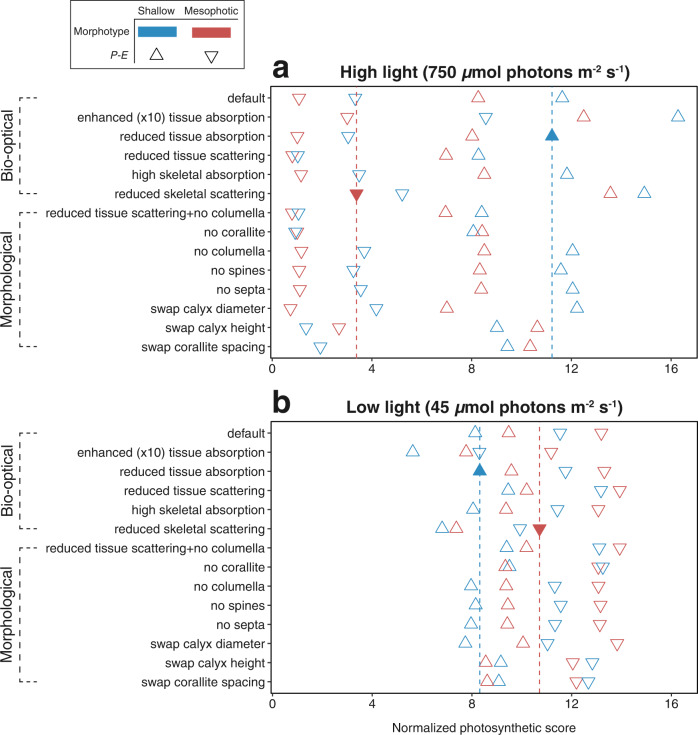
Photosynthetic scores normalized per tissue pixel of different bio-optical and morphological simulation scenarios. Photosynthetic scores normalized per tissue pixel of different bio-optical and morphological simulation scenarios under **a** high-light (equivalent to 5 m; 750 µmol photons m^−2^ s^−1^) and **b** low-light (equivalent to 50 m; 45 µmol photons m^−2^ s^−1^) conditions. Color denotes the morphotype (shallow: blue; mesophotic: red) and the ambient photosynthetic performance (*P-E*) is represented by shape (shallow: triangle point up; mesophotic: triangle point down). Filled triangles and dashed vertical lines represent the scores for settings as found in nature for shallow and mesophotic corals.

Based on the results of the morphometric analyses and the optical data from Kramer et al. (2022^31^; Tables S1-3), we performed a total of 112 optical simulations (Figs. 5 and 6; Figs. S2–S16). Generally, photosynthetic scores (*P;* see Methods) normalized per pixel tissue for high-incident irradiance simulations (750 µmol photons m^−2^ s^−1^) exhibited a wider range of values (*P* = 0.72–16.27) and displayed greater differences between shallow and mesophotic morphotypes than under low-light (45 µmol photons m^−2^ s^−1^) simulations (*P* = 5.64–13.94). The photosynthetic scores of shallow morphologies were dominated by an exponential decrease in fluence rate, while light attenuation was more homogenous for mesophotic corals (Fig. 5). Differences between morphotypes under all high-light scenarios (750 µmol photons m^−2^ s^−1^) were an order of magnitude higher in shallow versus mesophotic *P-E* performance inputs (*P* = 6.96–16.27 and 0.72–8.56, respectively; Fig. 6a). In contrast, regardless of the photosynthetic parameters (for both shallow and mesophotic), in nearly all simulation scenarios under low light the photosynthetic scores of mesophotic morphotypes consistently exceeded those of their shallow counterparts (by up to 30%; Fig. 6b). In most of the high-light simulation scenarios, shallow morphotypes exhibited 16–26% higher scores compared to the mesophotic morphotypes. For example, for the reduced tissue absorption scenario, photosynthetic scores were 40% higher for the shallow morphotypes under high-light (Fig. 6a; Fig. S5c–f). In contrast, for the low-light scenarios, photosynthetic scores were 15% higher for the mesophotic morphotypes (Fig. 6b; Fig. S5g–j).

In contrast to the patterns noted above, exchanging calyx height and corallite spacing values between shallow and mesophotic morphotypes moderately increased the photosynthetic scores for shallow morphotypes under low-light; whereas under high-light conditions there was no difference between morphotypes exhibiting the mesophotic *P-E* parameters. Removing the corallite resulted in similar photosynthetic scores for both shallow and mesophotic morphotypes under both light conditions. In addition, in most scenarios, surface rugosity was greater in shallow morphologies, which exhibited an up to twofold higher rugosity than their mesophotic congeners (Table S2). However, exchanging corallite spacing or height between the two morphotypes resulted in similar surface rugosities, which were akin to the mean value between the default morphotypes (Table S2). Lastly, since smaller skeletal features (e.g., the columella and the coenosteal spines) were shown to have a relatively minor impact on photosynthesis compared to the entire skeleton (e.g., Fig. S3 vs S10), as a test case, we examined the role of the columella when the tissue is mainly an absorbing medium, and the skeleton is mainly scattering (Fig. S6 vs S16).

## Discussion

Delineating the factors and functional traits that influence light capture by corals is fundamental for defining the range of light conditions under which survival, growth, and reproduction of a given coral species are possible. Using a mechanistic approach, we were able to uncover the role of key skeletal features of the coral *S. pistillata* in optimizing light harvesting. Our findings from models parameterized with morphological, optical, and photosynthetic data suggest that *S. pistillata’s* morphology influences light penetration within the coral tissue, demonstrating optimized photosynthesis of symbionts for local light conditions (i.e., shallow versus mesophotic).

The multivariate analysis pertaining to the small-scale morphological traits revealed distinct morphotypes between corals of shallow and mesophotic depths (Fig. 4). Three dominant traits were shown to drive divergence along the first PCoA axis: calyx diameter, theca height, and corallite marginal spacing, which varied between depths in a coordinated way: the increase in corallite marginal spacing with depth had a strong negative correlation with the decrease in corallite size, while the corallite centers maintained their relative location in reference to their neighboring corallites (Fig. 2a, h, i). Notably, we demonstrate that in shallow-growing colonies the corallites expand in both width and depth and are closely spaced, while the opposite occurs in mesophotic corals (Fig. 2a, b, i). In addition, we found that the coenosteal spines in mesophotic coral skeletons are significantly shorter and more closely spaced in comparison to those in the shallow depth (Fig. 2f). These findings are in line with earlier reports on the depth-related morphological changes in *S. pistillata*^29,44^. Similar to our own findings, Ow and Todd^8^ reported that the calices of shallow *Goniastrea pectinata* fragments were deeper and the septae were shorter than in deeper fragments. However, these patterns are not consistent in all hermatypic coral species, since each species displays a distinct morphology with varying dimensions of the different skeletal features between deep and shallow depths. For example, in *Dipsastraea speciosa* (formerly *Favia speciosa*) and *Diploastrea heliopora*, the corallites expand and deepen, but are more spaced under shallow-water conditions^45^; in *Galaxea facicularis*, corallite height increases and distance decreases with increasing light intensities, while corallite size increases under low-light levels^46^; and in *Montastrea cavernosa*, the corallites are smaller and more spaced in mesophotic corals, while septal length decreases in their shallower counterparts^47^. Taken together with our current findings, these reports indicate that variation in small-scale skeletal geometry across light regimes is species-specific. Consequently, one cannot draw generalized conclusions regarding shared skeletal features across coral species.

Presumably, adaptation to different light regimes involves light capture optimization on various spatial scales (e.g., colony and corallite levels), working in concert to enhance symbiont photosynthesis. Both coral tissue and coral skeleton can scatter light^48,49^, which can increase the probability of photon absorption by the coral’s symbiotic microalgae^50^. Previous studies have demonstrated the effectiveness of two-dimensional models for investigating the interaction between light and coral architecture on a colony scale^34,51^ and on a single corallite scale^8^. However, understanding how the different mechanisms of photoadaptation (e.g., morphological, physiological, and optical) interact to influence photosynthesis under a specific light regime is critical in determining the photic boundaries of any particular coral species. Integrating our morphometric results with recently obtained photosynthetic and optical data^31^, and using three-dimensional light propagation models, we applied a novel method by which to determine the functional significance of small-scale morphological traits with respect to the coral's internal irradiance distribution.

Our simulation results suggest that small-scale morphological traits influence *in-hospite* light distribution and absorption and thus affect coral photosynthesis. The change in the length-scale of morphological traits found within each of the two depth groups was shown to benefit the photosynthetic score (an approximation for photosynthesis; Eq. 1) with respect to their natural surrounding light regime (Figs. 5 and 6). Overall, samples from shallow depths exhibited a more rapid attenuation of light in the tissue and a greater ability to cope with excess light under high intensities, given that above the tissue surface the escaping flux (Φ) was enhanced by up to twofold higher from the incident irradiance (Fig. 5), thus supporting previous ecophysiological observations of light-adapted photosynthetic performance^31,33^. On the colony scale, Hoogenboom et al.^41^ found evidence of a strong reduction in energy available for coral growth under high-light levels and suggested that corals avoid the costs of excessive light exposure by means of altering colony morphology. Similarly, we show that the increase in corallite depth with increasing light intensities results in greater corallite self-shading, thus providing an effective mechanism for keeping irradiance within a photophysiologically optimal range (Fig. S17). In contrast, the mesophotic architecture exhibited a more spacious corallite structure, with a surface rugosity reduced by nearly twofold, which was advantageous in capturing low light. Hence, the combination of smaller, shallower, and more spaced corallites allowed for more light to be captured and utilized for photosynthesis (Figs. 2 and 5). This principle appears to be valid for light gradients within the colony itself, as recently shown by Drake et al.^73^: corallites exposed to more light (i.e., at the tip of the branch) were less spaced and larger than corallites at the base and junction of the branch. The greater space occupied by the coenosteum relative to the corallites, as documented for mesophotic-depth colonies, may reflect the host’s response to minimize light limitation for its photosymbionts. This response may reduce the denser pigmentation of the polyps, as the polyps reveal the largest pigmentation cross-section when all the tentacles are retracted^31,48^.

Surprisingly, simulations removing the corallites from the surface architecture yielded similar photosynthetic scores for the two morphotypes under the two light conditions (Fig. 6). Furthermore, surface rugosity was found to be similar for the two morphotypes when exchanging corallite spacing and height values (Table S2). This exchange moderately increased photosynthesis, i.e., promoting photosynthesis for shallow morphotypes under mesophotic light conditions, while the opposite occurred under shallow-water irradiance for mesophotic morphotypes. Several studies have described the important implications of coral structural complexity for light distribution^8,34,43,48^. In large-scale structures, variation in colony surface rugosity is related to competition and resource use, in which colonies whose surface distribution is complex have less light per unit surface area^43^. Similarly, a higher rugosity in small-scale structures increases self-shading^8,48,52^, as demonstrated in the shallow morphotypes of the present study. Consequently, we suggest that the corallite constitutes a dominant structural component, influencing surface rugosity and subsequently light harvesting. In contrast, skeletal features such as the columella and coenosteal spines were shown to have a relatively minor impact on photosynthesis compared to the entire corallite, potentially suggesting that their main role may be to provide additional structural and mechanical support to the coral tissue. It is possible, however, that different skeletal areas have different scattering properties^53,54^, which could potentially modulate the role of such small-scale features. In addition, such small-scale skeletal features might be especially beneficial for homogenizing irradiance distribution for densely absorbing and low scattering tissues (see example of columella in Figs. S6 and S16).

Typically, in comparison to shallow-water corals, corals in MCEs exhibit reduced growth rates^30,55^ and lower reproductive performances^56^, assumingly due to light being a limiting energy source. Our findings highlight that without specialized morphological modifications, light levels in MCEs would be insufficient to support the levels of photosynthesis required to sustain coral growth and reproduction (Fig. 6). Generally, too much light for corals would lead to photoinhibition; while too little light would be insufficient to supply the corals' nutrient demands. In terms of physiological adaptation, photosymbionts from shallow waters exhibit well-developed photo-protective mechanisms, such as high NPQ levels (i.e., higher excess energy dissipation) and increased antioxidant capacity, while the symbiotic microalgae residing in mesophotic corals make better use of low light^32,33^. However, light-driven physiological changes often occur in parallel with changes in host characteristics, since the *in hospite* light exposure of Symbiodiniaceae is highly dependent on tissue thickness, corallite rugosity, and tissue and skeleton optical properties^36,53,57^. A recent study, for example, found that light-enhancing mechanisms of the host's skeleton complement the photosynthetic demands of coral photosymbionts^31^. In corals, skeletal light scattering is modulated by varying the scale of skeleton-length structures, ranging from nanometers (e.g., CaCO3 nanograins) to millimeters (e.g., corallite)^58^. Hence, the skeleton geometry plays a vital role in dissipating adequate light to the tissue, as it influences the amount of energy that corals have available for growth and reproduction. Hoogenboom et al.^41^ posited that at the boundaries of the depth distribution, photoacclimation (i.e., physiological plasticity) cannot compensate for changes in morphology, and an adjustment of colony skeletal form appears to be the dominant phenotypic response; whereas photoacclimation is more important at intermediate depths. In line with that study, our optical simulations suggest that the host morphology can strongly affect the photosymbionts’ light environment of photosymbionts.

In addition to phenotypic plasticity, morphological variability can also result from genetic influences^59^. To date, only a few studies have examined depth-related genetic partitioning in coral populations, demonstrating distinct patterns of vertical connectivity among species^22,60,61^. Although our study species, *S. pistillata*, was previously found to belong to the same clade throughout its depth gradient in the Red Sea^44^, it does not necessarily exclude the possibility of genetic adaptation to depth. Apart from genetic influences, the smaller skeletal proportions in mesophotic corals may be a result of energy efficiency favoring reduced investment in skeletal features, arguably due to lower calcification rates^16,30^, rather than being exclusively adaptive responses to maximize light. Notwithstanding these energetic restraints, minimal energetic use is required to form the smaller mesophotic structures compared to the well-developed shallow architecture, since the need to create self-shading microhabitats is minimized in low-light environments.

Our results indicate that mesophotic *S. pistillata* skeletons exhibit a greater porosity in comparison to their shallow congeners (Fig. 3b; see Fig. 1b, c vs e, f). Corals growing under decreased pH levels usually exhibit increased porosity due to reduced calcification rates^62,63^. Similarly, the lower calcification rates of mesophotic *S. pistillata* colonies^30^ may explain their increased porosity. A recent study by Fordyce et al.^64^ examined whether the endolithic microbial communities in coral skeletons may benefit from higher colony porosity since this potentially makes more space available for colonization in skeletal pores. However, they conclude that light capture by endoliths is affected by the material properties of the skeleton (i.e., density) and not by its porosity. As shown by the µCT scans, the external engulfing-skeleton of mesophotic *S. pistillata* is thicker than the external engulfing-skeleton of shallow-water branches (Fig. 1b, c, e, f). Given the imperforate nature of *S. pistillata* (i.e., its tissue does not penetrate the skeleton), we suggest that porosity in *S. pistillata* may be negligible in regard to light acquisition capability. However, unlike *S. pistillata*, the porous skeleton of perforate-tissue species may have a more significant function in light capture due to their tissues intercalating through the skeletal framework. Thus, we encourage future research into this issue in other coral species.

Although light energy is the primary energy source in the shallow waters^65^, corals do not rely entirely on this form of energy. As mixotrophs, corals can also acquire energy from consuming zooplankton and particulate organic matter^66^. In shallow-water corals, heterotrophy can support survival during thermal stress by supplying energy to sustain symbiont autotrophy^67^, while in some mesophotic species, heterotrophy can provide the host with an alternate source of energy in the lack of light^68^. However, since corallites of mesophotic *S. pistillata* colonies are significantly smaller than in their shallow congeners (Fig. 2a), this could potentially limit the size range of zooplankton available for capture. Nevertheless, Martinez et al.^33^ have shown that the photosynthesis pathway is the main source of carbon in both shallow and mesophotic *S. pistillata*, while heterotrophy represents a lower but similar portion of the total energy budget for both depths. Since quantitative changes in energy sources along the depth gradient are only known for a limited number of depth-generalists, with the findings being species-specific^40^, the role of heterotrophy as an energetic strategy at mesophotic depths remains to be further explored.

Naturally, because the mesophotic light environment is significantly lower and generally cooler than the shallow reef, mesophotic colonies relatively experience fewer bleaching events than corals inhabiting shallow waters^69^. Since the corallite is a key component in light propagation, as suggested earlier, differences in bleaching susceptibility between morphotypes are also likely correlated to differences in corallite architecture rather than the coenosteum^53,58^. Previous findings show that the shallow corallites of *S. pistillata* exhibited greater scalar irradiance enhancement than mesophotic ones, and corallites of both morphotypes enhanced scalar irradiance compared to their respective coenosteum^31^. Consequently, the higher irradiance exposure in shallow-water morphotypes could precipitate a greater bleaching response within their more structurally complex corallites than mesophotic ones, due to the greater light enhancement^70^. However, morphotype alone is not a sufficient predictor for bleaching response under increased thermal stress (as predicted by the optical feedback loop hypothesis^70^), as photoinhibition within a given morphotype depends on the level of light exposure (Fig. 6) and other physiological factors. There is a great variability in the magnitude of skeleton scattering among colony and corallite morphology^53,58^, which warrants further work examining the bleaching susceptibility of morphotypes in different light environments.

In conclusion, our findings provide fundamental insights into how 3D small-scale skeletal coral designs and properties modulate photosynthesis. The novelty of these findings lies in the empirical demonstration of how morphology favors the use of light needed for photosynthesis, revealing a finely tuned photo-acquisition to local light. Our 3D light simulations have shown that regardless of the optical modifications, mesophotic coral morphological traits consistently promoted a more effective light acquisition for photosynthesis under low-light simulations; while shallow coral morphological traits were better structured to cope with the high-light intensities they encounter. These findings indicate that some morphological modifications constitute an essential component of photoacclimation at the photic boundaries. Moreover, coral populations living on the threshold of their optimal environment and adapted to extreme conditions have become useful models by which to predict the future functioning of coral reefs in light of climate change. Our 3D light models, integrating morphological and optical traits, could thus be applied to improve predictive models of coral responses to environmental changes.

## Materials and methods

### Coral sampling and preparation

The study was conducted at the coral reefs of the northern GoE/A, Red Sea. The scleractinian coral *Stylophora pistillata* was chosen as a model species for this study due to its importance as an eco-engineering species in the GoE/A. *S. pistillata* is a branching colony characterized by very small-immersed corallites arranged in a plocoid morph, exhibiting a solid style-like columella with six poorly developed septa, and a spiny coenosteum^71^. Furthermore, it exhibits a wide bathymetric distribution (0–60 m)^17,72^ and pronounced morphological variation in colony growth form with depth, from a subspherical densely branched form in the shallows to a more spread-out branch morphology in mesophotic environments (Fig. 1).

In February 2021, Fragments from intact adult coral colonies (ca. 20–25 cm in diameter) were collected during recreational and closed-circuit rebreather dives from shallow (4–5 m) and upper mesophotic (45–50 m) depths, corresponding to 40–45% and 3–8% of midday surface PAR, respectively^25^. In total, fragments from 30 colonies were used for this study (*n* = 15 per depth). Conspecific coral colonies were sampled at least five meters apart to avoid sampling clone mates, and fragments were sampled from the center of each colony to avoid within colony variation^73^. The samples were submerged in 5% sodium hypochlorite (NaClO) for 24 hours to dissolve the soft tissue, rinsed with distilled water, and air-dried at room temperature.

### X-ray microtomography and morphometrics

For analysis of the morphometric characters, each sample was scanned using high-resolution micro-computed tomography (μCT), conducted with a Nikon XT H 225ST µCT (Nikon Metrology Inc., USA) at The Steinhart Museum of Natural History, Tel Aviv University. *S. pistillata* specimens were scanned at an isotropic voxel (volume pixels) size of 10 μm (﻿360° rotation), with voltage and current set to 170 kV and 56 µA, respectively. Scans from each specimen were saved in a TIFF image format for 3D volume rendering and quantitative analysis using the software Dragonfly (© 2021 Object Research System (ORS) Inc.).

All measurements were taken from random intact corallites and from the coenosteum surrounding them, and which were not in a budding state nor at the colony margins (at least 2 cm from the distal branch tip to avoid areas of recent growth). A total of ten small-scale (mm) skeletal morphometric traits were measured (≥10 measurements per trait per sample; Fig. 1): calyx diameter (CD), theca (corallite wall) height (TH), septal length (SL), septal width (SW), columella height (CH), coenosteal spine spacing (SS), coenosteal spine length (SPL), coenosteal spine width (SPW), and corallite spacing, which was measured in two ways: the distance between neighboring corallite centers (CSC) and minimal distance between neighboring corallites (CSM). An additional measurement comprised branch thickness (mm). All skeletal metrics were perpendicularly aligned to the sample’s growth axis prior to measurement. Lastly, apparent porosity was determined as the percentage ratio of pore volume to the total volume occupied by the coral skeleton.

### 3D light propagation models

To model the effect of different skeletal features on light capture we developed a 3D Monte Carlo simulation^50,74^. Monte Carlo Simulations are probability distribution models that are widely used, validated, and accepted for modeling light propagation in biological tissues and often considered the gold standard for modeling complex tissue architectures^75^. Detailed explanations of the core simulation process can be found in Wang et al.^76^. Briefly, photons are launched through a tissue with independent absorption and scattering centers, and interact with the tissue via a random process of light scattering and absorption. The overall probability of absorption and scattering are based on the inherent optical properties of the tissues of interest, yielding a characteristic, average light distribution. The optical properties considered in the simulation were absorption coefficient (µa), scattering coefficient (µs), and anisotropy of scattering (g). Monte Carlo Simulations allow for modeling any source geometry, with mesh-based and voxel-based methods existing for modeling complex 3D geometries.

### Source architecture

We used the average morphological parameters obtained from µCT scanning to create representative coral skeleton designs for shallow and mesophotic corals (Fig. 1 and Table S1). For the coral tissue, we assumed thicknesses based on previous measurements^31^. We acknowledge that under natural conditions coral tissues are flexible, and expansion and contraction can affect light propagation. However, for simplicity, we assumed here only the contracted tissue state, comprising one continuous tissue type with average optical properties (see below). The tissue covering the coenosteum was set to the maximal length of the coenosteal spines, and filled the calyx cavity to mimic a fully contracted coral polyp. The void space was filled with seawater.

### Simulation settings

To systematically test the role of different coral morphologies on coral light transport, we designed a large number of simulations with varying morphologies and optical properties. As our primary aim was to understand the role of differences in the small-scale morphology between shallow and mesophotic morphotypes, independent of other differences between these groups that can affect light propagation (e.g., changes in algal density and/or skeletal scattering), we ran simulations under identical optical properties for both morphotypes. In addition, to understand whether differences in light propagation between morphotypes remain true for various optical properties, we systematically tested scenarios where we varied tissue pigmentation, tissue scattering, skeletal scattering, and skeletal absorption one at a time. ‘Default’ optical properties (simulation scenario “1”) were chosen based on previous studies^31,77^, and assumes high skeletal scattering (*µ*_*s*_*’ skeleton* = 15 cm^−1^), intermediate tissue scattering (*µ*_*s*_*’* tissue = 10 cm^−1^) as well as moderate tissue pigmentation/absorption (*µ*_*a*_ tissue = 1.18 cm^−1^) and low skeletal absorption (*µ*_*a*_ skeleton = 0.01 cm^−1^). We then systematically varied one optical property at a time. Simulation scenario “2” reduced pigmentation—uses a tissue absorption coefficient that is about half of the default value; scenario “3” Enhanced pigmentation—uses ×10 enhanced *µ*_*a*_ tissue; scenario “4” low *µ*_*s*_*’* tissue—uses ×10 lowered *µ*_*s*_*’* tissue; scenario “5” High *µ*_*a*_ skeleton—uses high skeletal absorption; and scenario “6” low *µ*_*s*_*’* skeleton—uses ×5 lowered µs*’* skeleton. While these additional simulations are by no means thought to be exhaustive, they should be regarded as representative of different optical properties this coral could have in nature resulting from adaptation to different environments.

The scattering anisotropy and the phase function were assumed to be constant (*g*-value = 0.9 for both tissue and skeleton; see ref. ^50^). The Monte Carlo model assumes the Henyey-Greenstein (HG) phase function which has been routinely used for various types of human tissue and in a few coral studies. A recent study used inverse Optical Coherence Tomography and suggested that the use of the HG phase function might be inappropriate for some corals and some regions of corals (specifically coral tissue^4^). However, so far it is unknown whether this might also be the case for different corals, such as *S. pistillata* used in this study, and thus should be investigated in future studies.

The contribution of individual key architectural features were assessed using a “knock-out” procedure, which involves removing one morphological trait at a time and assessing the light distribution over the entire coral architecture. For the simulation in which the calyx was removed, we kept the tissue volume constant by redistributing the tissue over the coenosteum. We further quantified the effects of morphological traits on surface area (mm^2^), surface rugosity (geometric surface area divided by real surface area), and tissue volume (mm^3^) for shallow and mesophotic architectures. Moreover, we also exchanged the mean measurement values of the above traits between shallow and mesophotic morphotypes to further test their functionality. Finally, we examined the contribution of the skeletal architecture given the same optical properties, with each simulation scenario focusing on one modified optical trait.

Since the application of microsensors is affected by the optical response of the spherical microsensor, the tip size, and the ability to manually perform measurements within the complex corallite structure of the small corallite features of *S. pistillata* (compared to other coral species with larger corallites) is extremely challenging, we assumed that within each coral component (i.e., tissue and skeleton) the optical properties were homogenous. So far, optical properties of *S. pistillata* from shallow and mesophotic environments have only been determined via diffuse reflectance spectroscopy (see Kramer et al. (2022; Table S3)) which only facilitates an estimate of bulk optical properties for tissue and skeletal compartments. In addition, to characterize cell and chlorophyll-*a* densities, we airbrushed the entire coral fragment which pools potential spatial variability in tissue pigmentation. To include potential spatial variability in tissue pigmentation, imaging-based approaches could be used to approximate differences in tissue absorptivity^78^.

For simplicity, we modeled the angle of sunlight to be parallel rays perpendicular to the coral surface, although the light angle is not fixed under natural conditions. Future studies based on our model could aim to incorporate more complex models that include multiple light angles.

To determine the optimal simulation time, we executed multiple tests with the same setting and varying simulation times. We found that simulations over two hours yielded similar results to those of the two-hour simulations, and thus decided to use two-hour simulations in all scenarios. With this setup, we executed the MC simulation code (2 h/ ~5 × 10^7^ photons; resolution = 0.005 mm/pixel) and obtained the fluence rate information on the 3D coral models.

### 3D photosynthesis model

To evaluate the relationship between coral light capture and coral photosynthesis we developed a 3D photosynthesis model. The model uses the volumetric fluence rate distribution to calculate a tissue photosynthesis approximation for complex coral architectures at a high spatial resolution. We developed a script to calculate a ‘relative photosynthesis score’ using our experimentally determined photosynthesis-irradiance (*P-E*) data from Pulse Amplitude Modulation (PAM) chlorophyll-*a* fluorometer (Fig. S17), and the following relationship^79^ (Table S1):

1 $$P={P}_{{\max }}\frac{E}{{E}_{{{{{{\rm{opt}}}}}}}}{e}^{1-\frac{E}{{E}_{{{{{{\rm{opt}}}}}}}}}$$

where *P* represents the relative gross photosynthesis score, *E* is the fluence rate, ${P}_{{\max }}$ represents the maximum gross photosynthesis rate, and ${E}_{{{{{{\rm{opt}}}}}}}$ is the optimal fluence rate at ${P}_{{\max }}$. Score values were normalized by tissue voxels for each morphotype. We note that actual rates of volumetric photosynthesis can be affected by tissue absorptivity (i.e., algal density and pigmentation). In order to assess the importance of morphological changes independent of such pronounced effects on volumetric O_2_ evolution, tissue absorptivity was assumed to be equal for shallow and mesophotic morphotypes for all optical simulations (see Text S1). Relative electron transport rate was used to describe PSII photochemical efficiency, due to their rapid, non-invasive approach. The advantage of this photosynthesis model is that it assumes *in hospite* irradiance values, and not the commonly used incident downwelling irradiance values, which are very different from *in hospite* scalar irradiance values^48^. For each experimental setting, we calculated the actual fluence rates based on the in-situ light levels in February^25^: 750 and 45  µmol photons m^−2^ s^−1^ for shallow (5 m) and mesophotic (50  m) depths, respectively.

### Statistics and reproducibility

Statistical analyses were performed using the R software^80^. Since in most cases the data did not conform to parametric test assumptions, intraspecies variations between depths for each morphological character were tested using a mixed-effects permutational analysis (MEPA; 999 permutations) and included the sample ID as a random effect. These analyses were run using the {lme4}^81^ and {predictmeans}^82^ packages. A principal coordinates analysis (PCoA) based on a Euclidean distance matrix of standardized data was created with the {vegan} package to visualize the pattern of morphological variation between depths in a multivariate trait space. Permutational multivariate analysis of variance (PERMANOVA; 999 permutations) was performed to determine the overall effect of depth on the morphological patterns. Traits were highlighted as important for a given axis based on whether their loadings exceeded the null contribution value of 10% (100% divided by ten variables). Pearson’s correlation coefficients were used to assess pairwise correlations among the different skeletal traits. Similarity percentage analysis (SIMPER) was conducted to determine which morphological traits were responsible for most of the variation between depths^83^.

### Reporting summary

Further information on research design is available in the Nature Research Reporting Summary linked to this article.

## Supplementary information

Supplemental Material

Reporting Summary

## Data Availability

All the datasets generated and/or analyzed during the current study are available in the Dryad digital repository^84^.

## Code availability

All the code scripts used for this study are available in the Dryad digital repository^84^.
