# Supplementary material for: Morpho-functional traits of the coral Stylophora pistillata enhance light capture for photosynthesis at mesophotic depths
Source: Commun Biol. 2022 Aug 24;5:861. doi: 10.1038/s42003-022-03829-4 (PMC9402581; doi:10.1038/s42003-022-03829-4)
Supplement: Supplementary file 1 — Supplemental Material [file 42003_2022_3829_MOESM1_ESM.pdf]

Supplementary for

Morpho-functional traits of the coral Stylophora pistillata enhance light capture for photosynthesis at mesophotic depths

Netanel Kramer[1*], Jiaao Guan[2], Shaochen Chen[3], Daniel Wangpraseurt[3,4], Yossi Loya[1]

[1] *School of Zoology, Faculty of Life Sciences, Tel-Aviv University, Tel Aviv, Israel*

[2] *Department of Electrical and Computer Engineering, University of California San Diego, San Diego, USA*

[3] *Department of Nanoengineering, University of California San Diego, San Diego, USA*

[4] *Scripps Institution of Oceanography, University of California San Diego, San Diego, USA*

* Corresponding author: Netanel Kramer <nati.kramer@gmail.com>

Table S1. Summary table of the average morphological traits (in mm) and photosynthetic light response (*P*-*E*) parameters used in the light simulation models (resolution = 0.005 mm/pixel).

Trait	Shallow	Mesophotic
Morphology		
Tissue thickness	0.12	0.07
Calyx diameter	0.98	0.62
Corallite marginal spacing	0.50	0.80
Theca height	1.20	0.84
Columella height	0.64	0.61
Septal length	0.20	0.13
Septal width	0.12	0.08
Coenosteum spine length	0.16	0.10
Coenosteum spine width	0.10	0.08
Coenosteum spine spacing	0.15	0.13
P-E		
Maximum photosynthetic rate (P_{max})	22.2	19.6
Saturation irradiance (E_k)	120.0	64.4
Optimum irradiance (E_{opt})	326.2	175.1

Table S2. The surface area (mm^2), surface rugosity (real surface area divided by geometric surface area divided; more complex < 1), and tissue volume (mm^3) for shallow and mesophotic architectures for different morphological scenarios.

	**Morphotype**	***In-situ***	**No calyx**	**No columella**	**No coenosteal spines**	**No septae**	**Corallite spacing exchanged**	**Corallite diameter exchanged**	**Corallite height exchanged**
Surface area	Shallow	8.93	3.72	8.79	7.87	6.44	10.58	6.40	7.18
(mm^2)	Mesophotic	5.27	2.80	5.15	4.59	4.16	4.25	7.52	6.38
Surface	Shallow	4.08	1.70	4.01	3.59	2.94	3.34	5.10	3.28
rugosity	Mesophotic	2.62	1.39	2.55	2.28	2.06	3.38	2.37	3.17
Tissue volume	Shallow	0.92	0.22	0.96	0.95	1.09	1.02	0.31	0.68
(mm^3)	Mesophotic	0.31	0.12	0.32	0.32	0.36	0.26	0.73	0.39

Table S3. Summary table of the light simulation scenarios containing the architectural (Table S1; in mm) and optical parameters (Kramer et al. 2021; per cm^{-1}) used in each scenario. These parameters were applied in the models of (**a**) shallow and (**b**) mesophotic morphotypes under both low-light (45 µmol photons m^{-2} s^{-1}) and high-low (750 µmol photons m^{-2} s^{-1}) conditions. Values in bold indicate the changed value for simulation from default settings.

**Simulation scenario**	**a. Shallow morphotype**								
	**Calyx (mm)**	**Theca (mm)**	**Columella (mm)**	**Spines (mm)**	**corallite spacing (mm)**	μ_a **tissue**	μ_a **skeleton**	μ_s**' tissue**	μ_s**'** ***skeleton***
Default	0.98	1.2	0.64	0.16	0.5	1.18	10	0.01	15
Reduced tissue absorption	0.98	1.2	0.64	0.16	0.5	**0.66**	10	0.01	15
Enhanced (x10) tissue absorption	0.98	1.2	0.64	0.16	0.5	**11.8**	10	0.01	15
Reduced tissue scattering	0.98	1.2	0.64	0.16	0.5	1.18	**0.1**	0.01	15
High skeleton absorption	0.98	1.2	0.64	0.16	0.5	1.18	10	**0.47**	15
Reduced skeleton scattering	0.98	1.2	0.64	0.16	0.5	1.18	10	0.01	**3**
No calyx	**0**	**0**	**0**	0.16	0.5	1.18	10	0.01	15
No columella	0.98	1.2	**0**	0.16	0.5	1.18	10	0.01	15
No coenosteal spines	0.98	1.2	0.64	**0**	0.5	1.18	10	0.01	15
Exchange calyx diameter	**0.62**	1.2	0.64	0.16	0.5	1.18	10	0.01	15
Exchange calyx height	0.98	**0.84**	0.64	0.16	0.5	1.18	10	0.01	15
Exchange corallite spacing	0.98	1.2	0.64	0.16	**0.8**	1.18	10	0.01	15

Simulation scenario	b. Mesophotic morphotype								
	Calyx (mm)	Theca (mm)	Columella (mm)	Spines (mm)	corallite spacing (mm)	μ_a tissue	μ_a skeleton	μ_s' tissue	μ_s' *skeleton*
Default	0.62	0.84	0.61	0.1	0.8	1.18	10	0.01	15
Reduced tissue absorption	0.62	0.84	0.61	0.1	0.8	**0.66**	10	0.01	15
Enhanced (x10) tissue absorption	0.62	0.84	0.61	0.1	0.8	**11.8**	10	0.01	15
Reduced tissue scattering	0.62	0.84	0.61	0.1	0.8	1.18	**0.1**	0.01	15
High skeleton absorption	0.62	0.84	0.61	0.1	0.8	1.18	10	**0.47**	15
Reduced skeleton scattering	0.62	0.84	0.61	0.1	0.8	1.18	10	0.01	**3**
No calyx	**0**	**0**	**0**	0.1	0.8	1.18	10	0.01	15
No columella	0.62	0.84	**0**	0.1	0.8	1.18	10	0.01	15
No coenosteal spines	0.62	0.84	0.61	**0**	0.8	1.18	10	0.01	15
Exchange calyx diameter	**0.98**	0.84	0.61	0.1	0.8	1.18	10	0.01	15
Exchange calyx height	0.62	**1.2**	0.61	0.1	0.8	1.18	10	0.01	15
Exchange corallite spacing	0.62	0.84	0.61	0.1	**0.5**	1.18	10	0.01	15

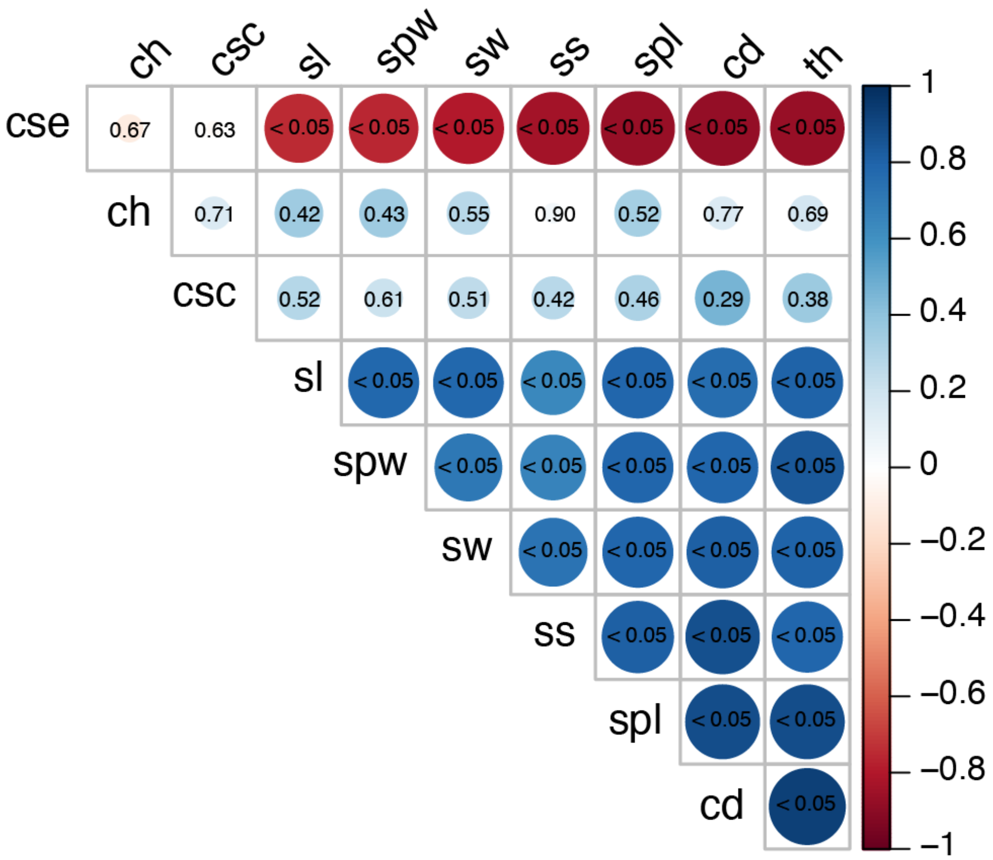

Figure S1. A correlation matrix based on Pearson's correlation coefficients between skeletal traits. Color scale denotes positive (*blue*) to negative (*red*) correlation coefficients. Significance corresponds to circle size and is shown as p-values within the circles.

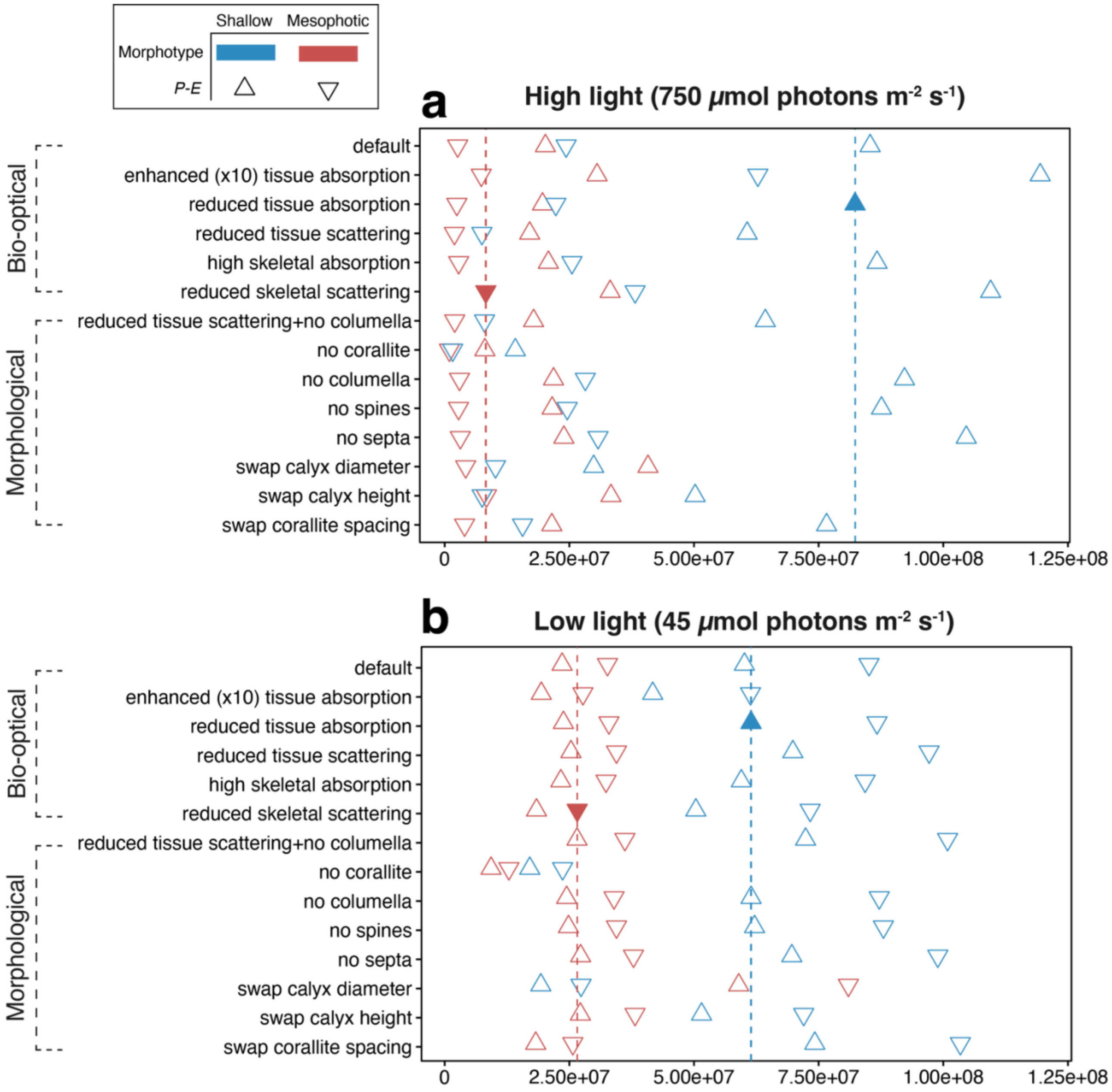

Figure S2. The total photosynthetic scores (summed by tissue pixel) of different bio-optical and morphological simulation scenarios under **(a)** high-light (equivalent to 5 m; 750 μmol photons m^{-2} s^{-1}) and **(b)** low-light (equivalent to 50 m; 45 μmol photons m^{-2} s^{-1}) conditions. Color denotes the morphotype (shallow: *blue*; mesophotic: *red*) and the ambient photosynthetic performance (*P-E*) is represented by shape (shallow: triangle point up; mesophotic: triangle point down). Filled triangles and dashed vertical lines represent the scores for settings as found in nature for shallow and mesophotic corals.

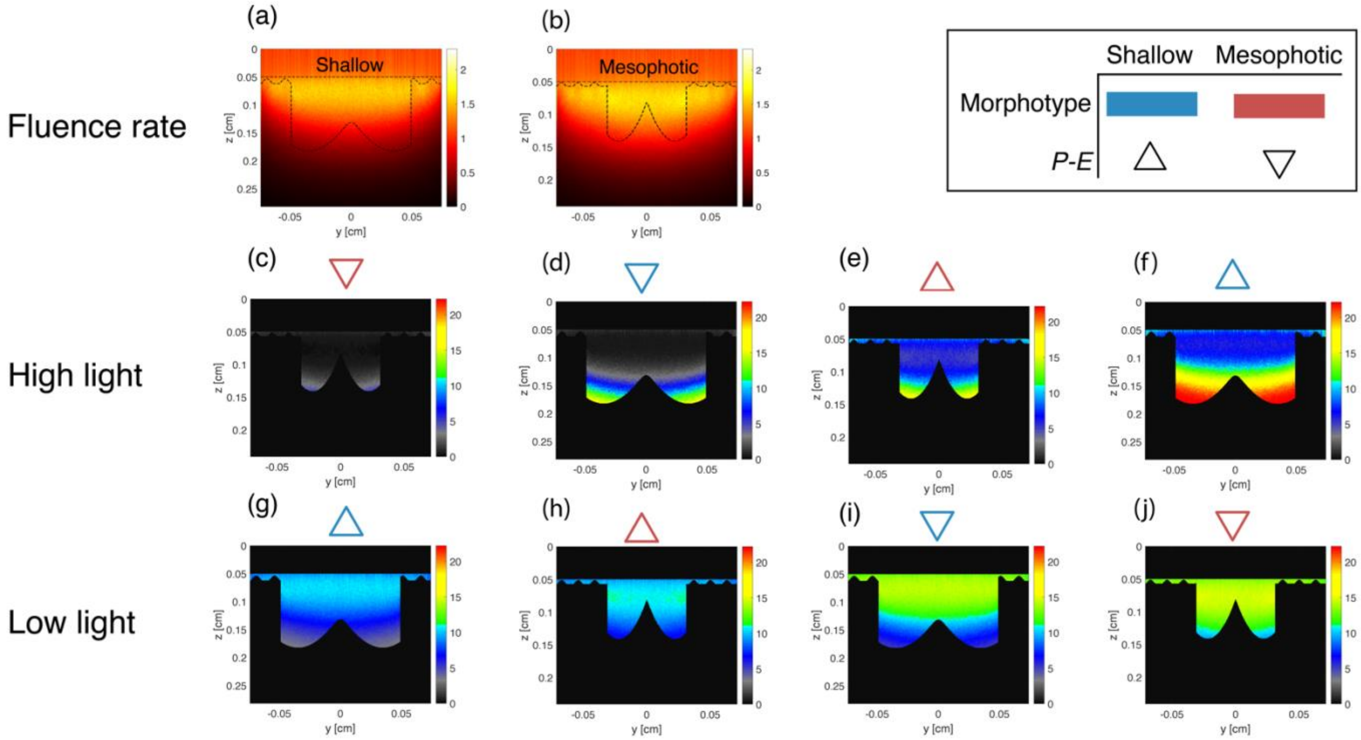

Figure S3. “Default settings” scenario (see Table S3). Light propagation simulations shown in 2D (y-z axes) under light intensities of (**c-f**) 750 (high light) and (**g-j**) 45 (low light) µmol photons m^{-2} s^{-1}. **(a-b)** Relative fluence rates (delivered as W m^{-2}; “fire” color gradient) with contour indicating the surface boundaries. **(c-j)** Photosynthetic score (“rainbow” color gradient) on the tissue layers. Triangle color above the figures denotes the morphotype (shallow: *blue*; mesophotic: *red*), and the ambient photosynthetic performance (*P-E*) is represented by shape (shallow: triangle point up; mesophotic: triangle point down).

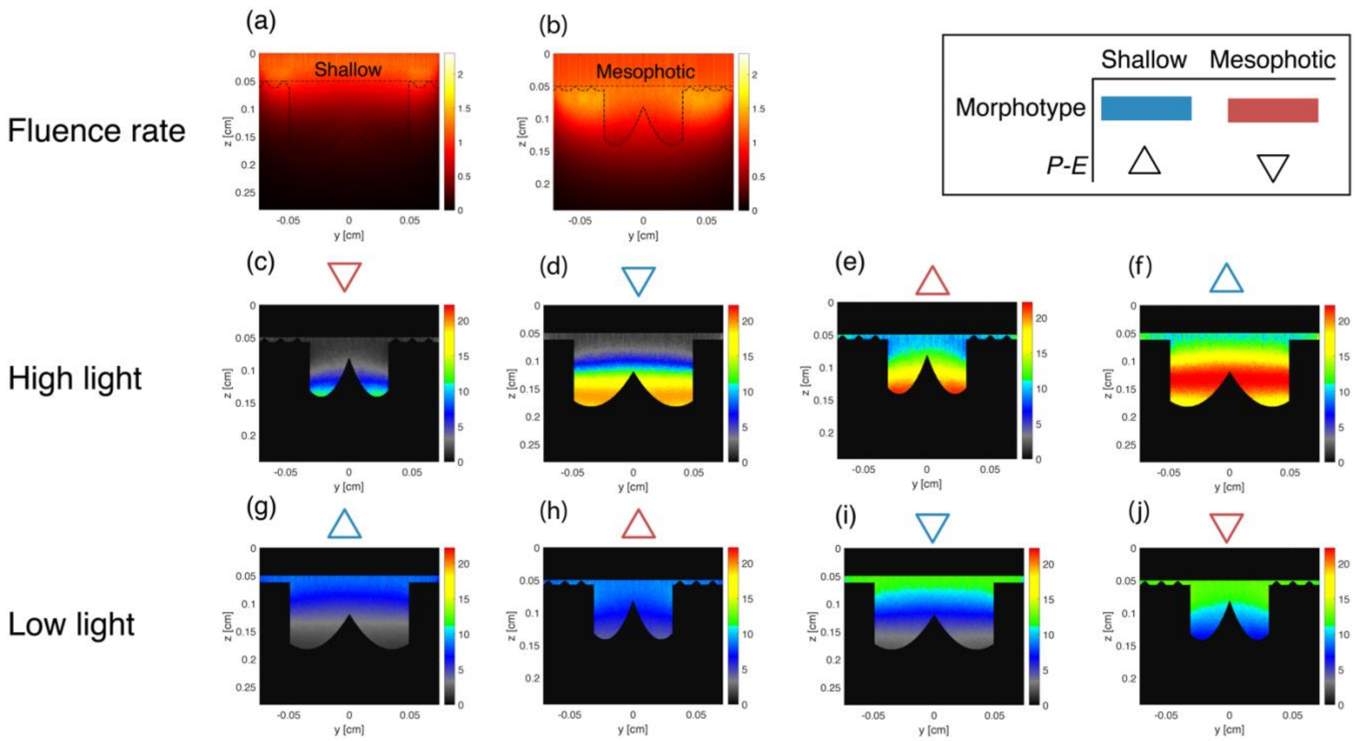

Figure S4. "Enhanced (x10) tissue absorption" scenario. Light propagation simulations shown in 2D (y-z axes) under light intensities of (**c-f**) 750 (high light) and (**g-j**) 45 (low light) μmol photons m^{-2} s^{-1}. **(a-b)** Relative fluence rates (delivered as W m^{-2}; "fire" color gradient) with contour indicating the surface boundaries. **(c-j)** Photosynthetic score ("rainbow" color gradient) on the tissue layers. Triangle color above the figures denotes the morphotype (shallow: *blue*; mesophotic: *red*), and the ambient photosynthetic performance (*P-E*) is represented by shape (shallow: triangle point up; mesophotic: triangle point down).

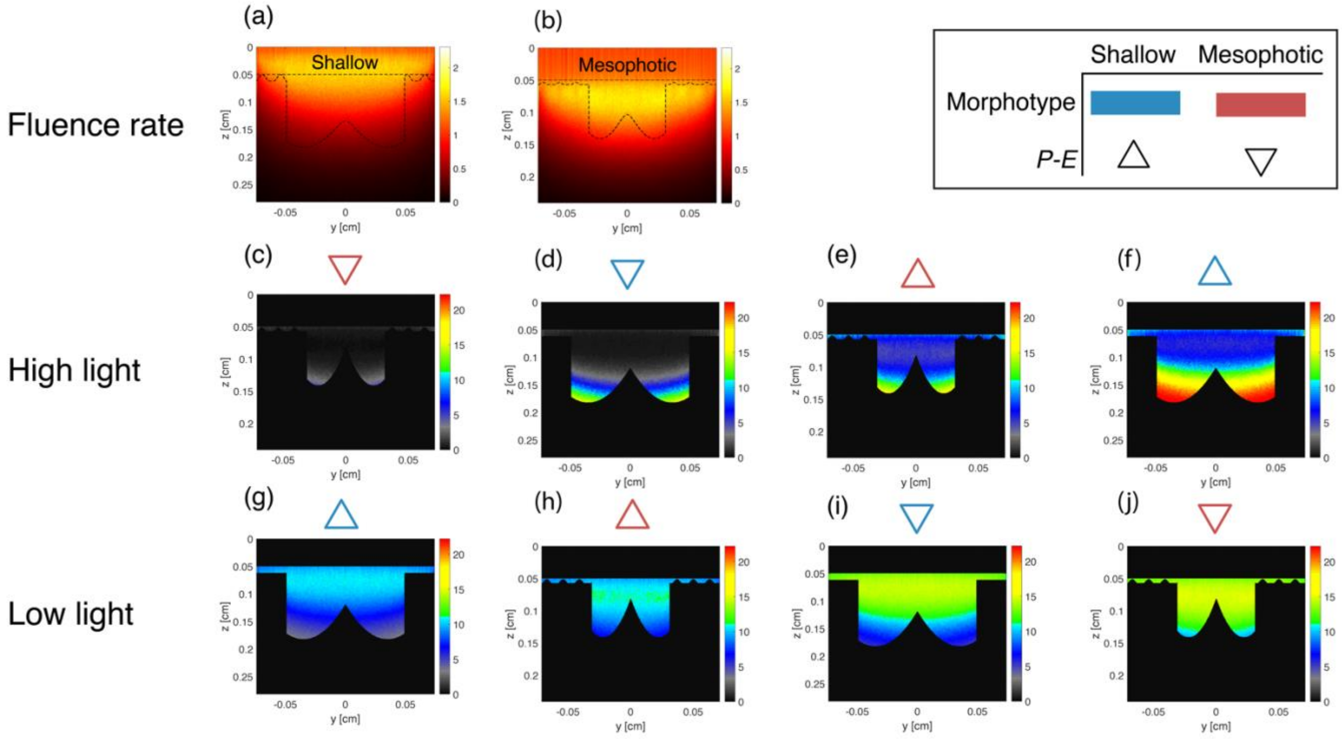

Figure S5. “Reduced tissue absorption” scenario. Light propagation simulations shown in 2D (y-z axes) under light intensities of (**c-f**) 750 (high light) and (**g-j**) 45 (low light) µmol photons m^{-2} s^{-1}. **(a-b)** Relative fluence rates (delivered as W m^{-2}; “fire” color gradient) with contour indicating the surface boundaries. **(c-j)** Photosynthetic score (“rainbow” color gradient) on the tissue layers. Triangle color above the figures denotes the morphotype (shallow: *blue*; mesophotic: *red*), and the ambient photosynthetic performance (*P-E*) is represented by shape (shallow: triangle point up; mesophotic: triangle point down).

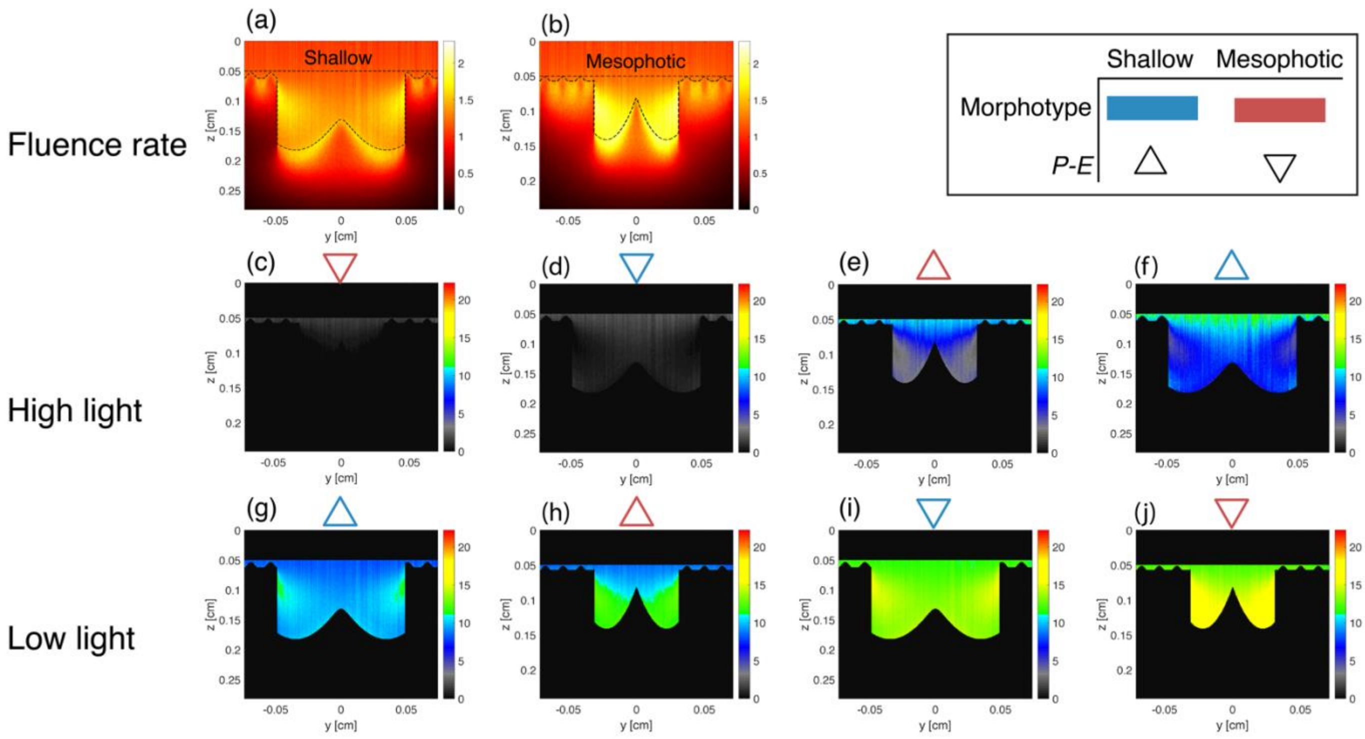

Figure S6. “Reduced tissue scattering” scenario. Light propagation simulations shown in 2D (y-z axes) under light intensities of (**c-f**) 750 (high light) and (**g-j**) 45 (low light) µmol photons m^{-2} s^{-1}. **(a-b)** Relative fluence rates (delivered as W m^{-2}; “fire” color gradient) with contour indicating the surface boundaries. **(c-j)** Photosynthetic score (“rainbow” color gradient) on the tissue layers. Triangle color above the figures denotes the morphotype (shallow: *blue*; mesophotic: *red*), and the ambient photosynthetic performance (*P-E*) is represented by shape (shallow: triangle point up; mesophotic: triangle point down).

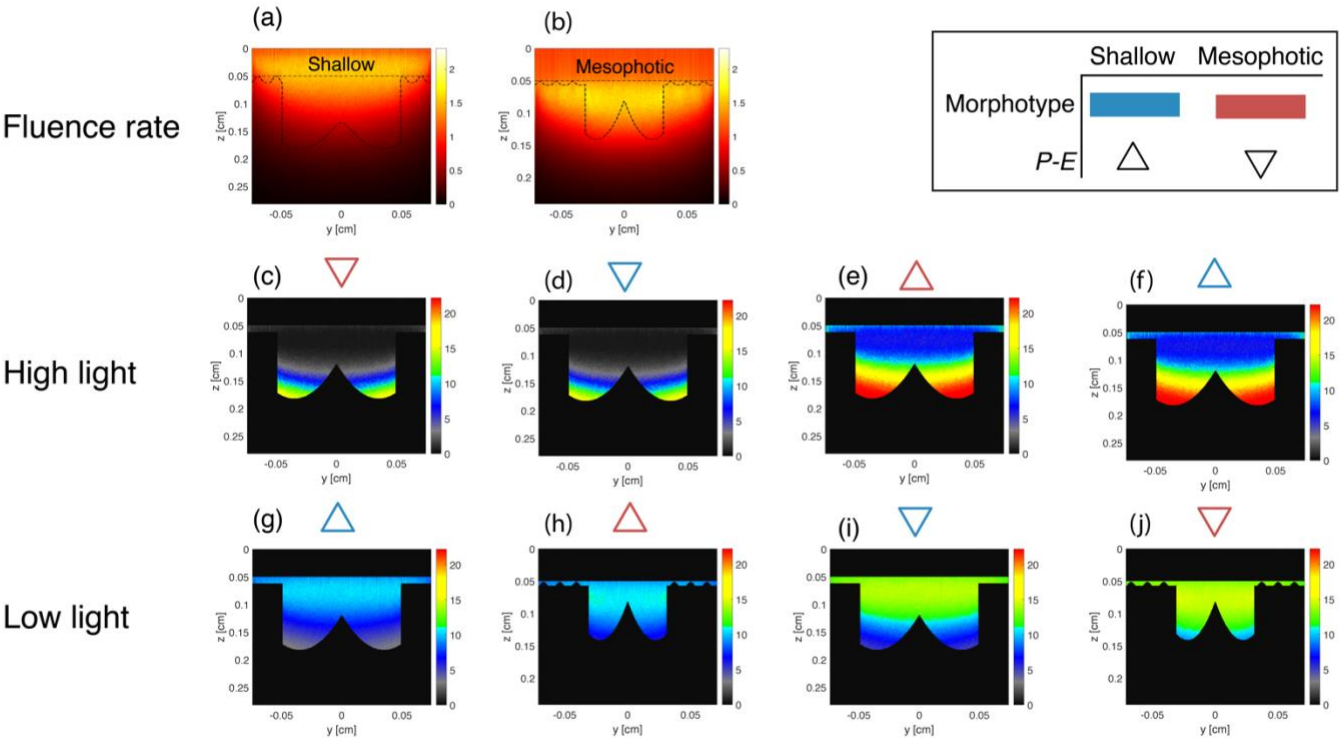

Figure S7. "High skeleton absorption" scenario. Light propagation simulations shown in 2D (y-z axes) under light intensities of (**c-f**) 750 (high light) and (**g-j**) 45 (low light) μmol photons m^{-2} s^{-1}. **(a-b)** Relative fluence rates (delivered as W m^{-2}; "fire" color gradient) with contour indicating the surface boundaries. **(c-j)** Photosynthetic score ("rainbow" color gradient) on the tissue layers. Triangle color above the figures denotes the morphotype (shallow: *blue*; mesophotic: *red*), and the ambient photosynthetic performance (*P-E*) is represented by shape (shallow: triangle point up; mesophotic: triangle point down).

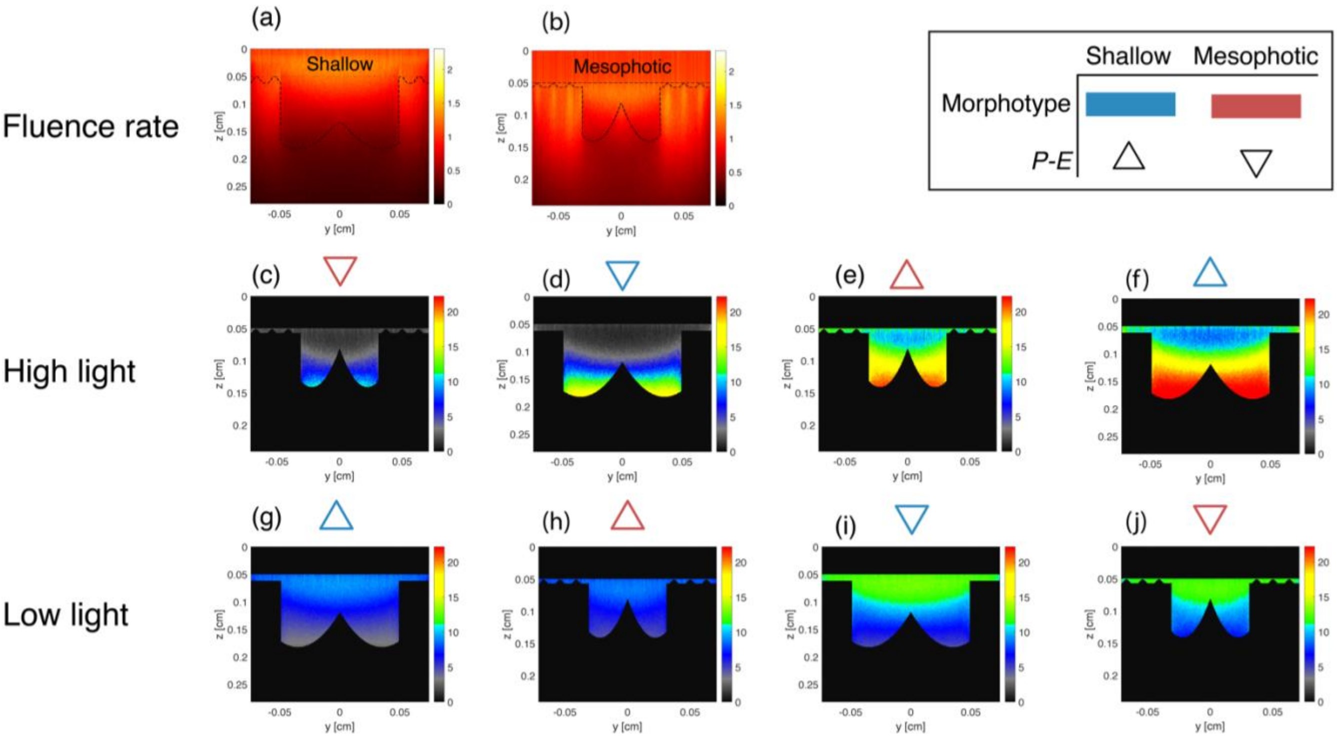

Figure S8. "Low skeleton scattering" scenario. Light propagation simulations shown in 2D (y-z axes) under light intensities of (**c-f**) 750 (high light) and (**g-j**) 45 (low light) μmol photons m^{-2} s^{-1}. **(a-b)** Relative fluence rates (delivered as W m^{-2}; "fire" color gradient) with contour indicating the surface boundaries. **(c-j)** Photosynthetic score ("rainbow" color gradient) on the tissue layers. Triangle color above the figures denotes the morphotype (shallow: *blue*; mesophotic: *red*), and the ambient photosynthetic performance (*P-E*) is represented by shape (shallow: triangle point up; mesophotic: triangle point down).

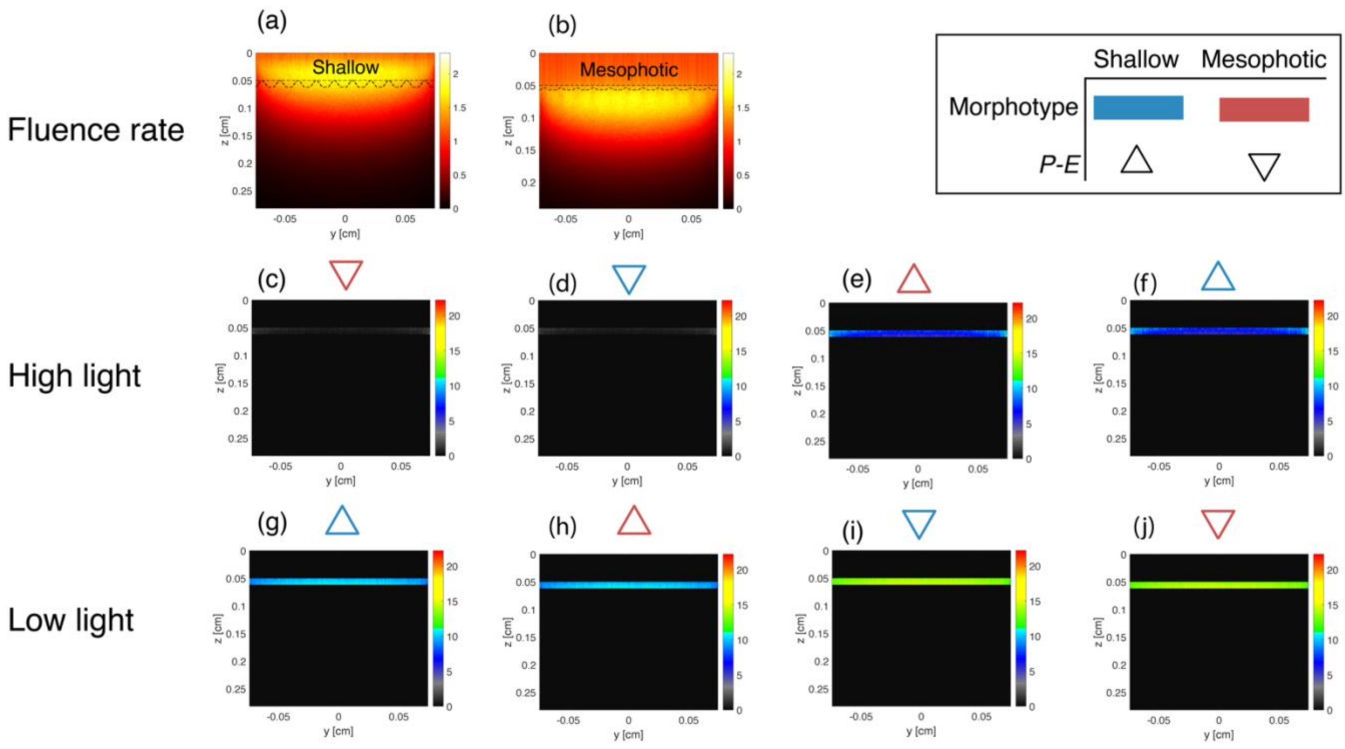

Figure S9. "No corallite" scenario. Light propagation simulations shown in 2D (y-z axes) under light intensities of (**c-f**) 750 (high light) and (**g-j**) 45 (low light) µmol photons m^{-2} s^{-1}. **(a-b)** Relative fluence rates (delivered as W m^{-2}; "fire" color gradient) with contour indicating the surface boundaries. **(c-j)** Photosynthetic score ("rainbow" color gradient) on the tissue layers. Triangle color above the figures denotes the morphotype (shallow: *blue*; mesophotic: *red*), and the ambient photosynthetic performance (*P-E*) is represented by shape (shallow: triangle point up; mesophotic: triangle point down).

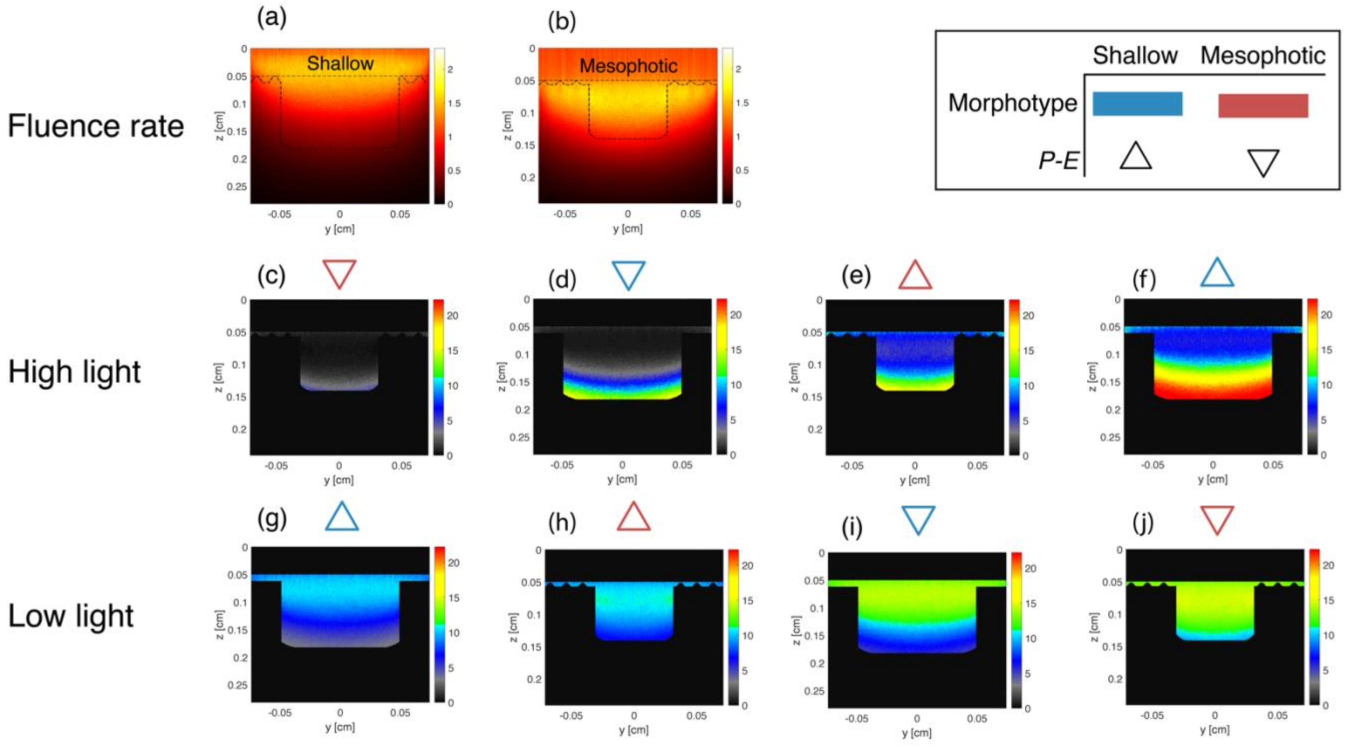

Figure S10. "No columella" scenario. Light propagation simulations shown in 2D (y-z axes) under light intensities of (**c-f**) 750 (high light) and (**g-j**) 45 (low light) µmol photons m^{-2} s^{-1}. **(a-b)** Relative fluence rates (delivered as W m^{-2}; "fire" color gradient) with contour indicating the surface boundaries. **(c-j)** Photosynthetic score ("rainbow" color gradient) on the tissue layers. Triangle color above the figures denotes the morphotype (shallow: *blue*; mesophotic: *red*), and the ambient photosynthetic performance (*P-E*) is represented by shape (shallow: triangle point up; mesophotic: triangle point down).

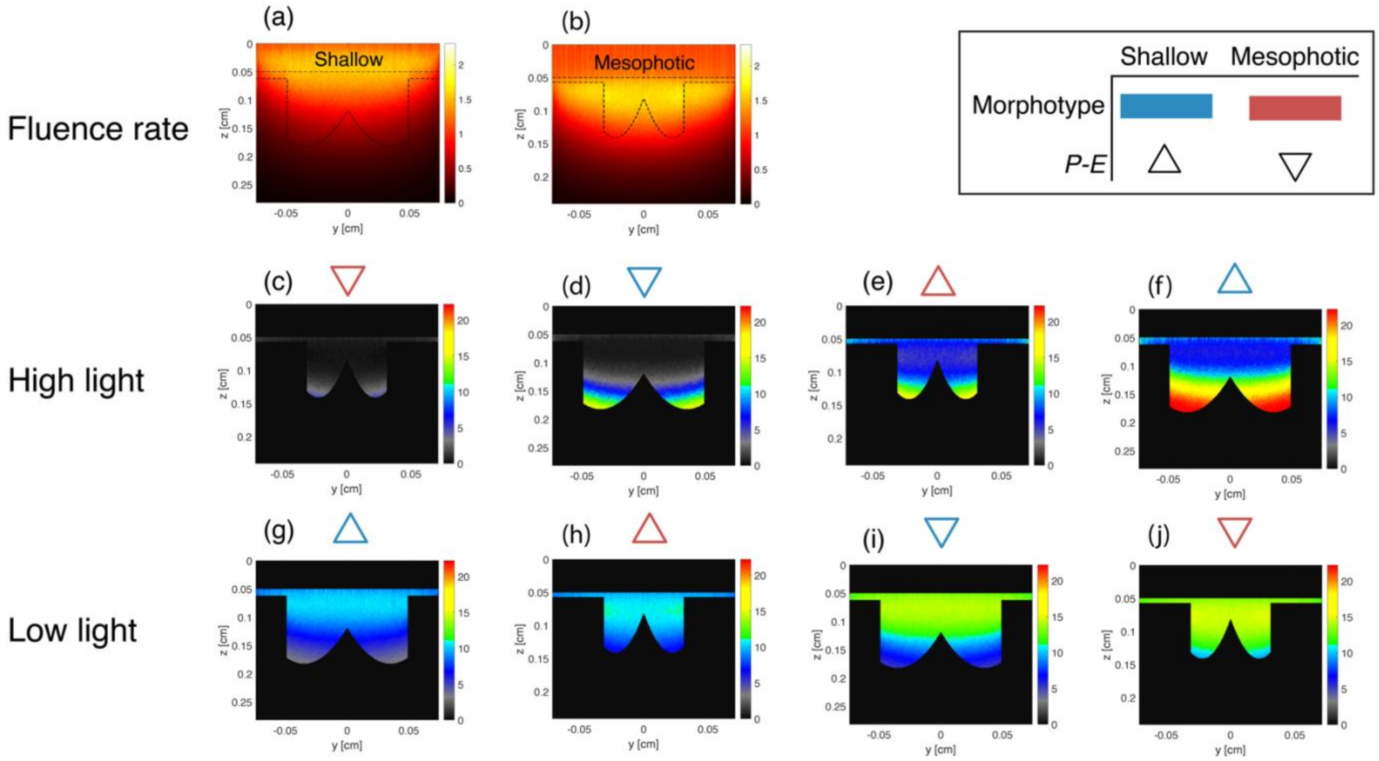

Figure S11. "No coenosteal spines" scenario. Light propagation simulations shown in 2D (y-z axes) under light intensities of (**c-f**) 750 (high light) and (**g-j**) 45 (low light) µmol photons m^{-2} s^{-1}. **(a-b)** Relative fluence rates (delivered as W m^{-2}; "fire" color gradient) with contour indicating the surface boundaries. **(c-j)** Photosynthetic score ("rainbow" color gradient) on the tissue layers. Triangle color above the figures denotes the morphotype (shallow: *blue*; mesophotic: *red*), and the ambient photosynthetic performance (*P-E*) is represented by shape (shallow: triangle point up; mesophotic: triangle point down).

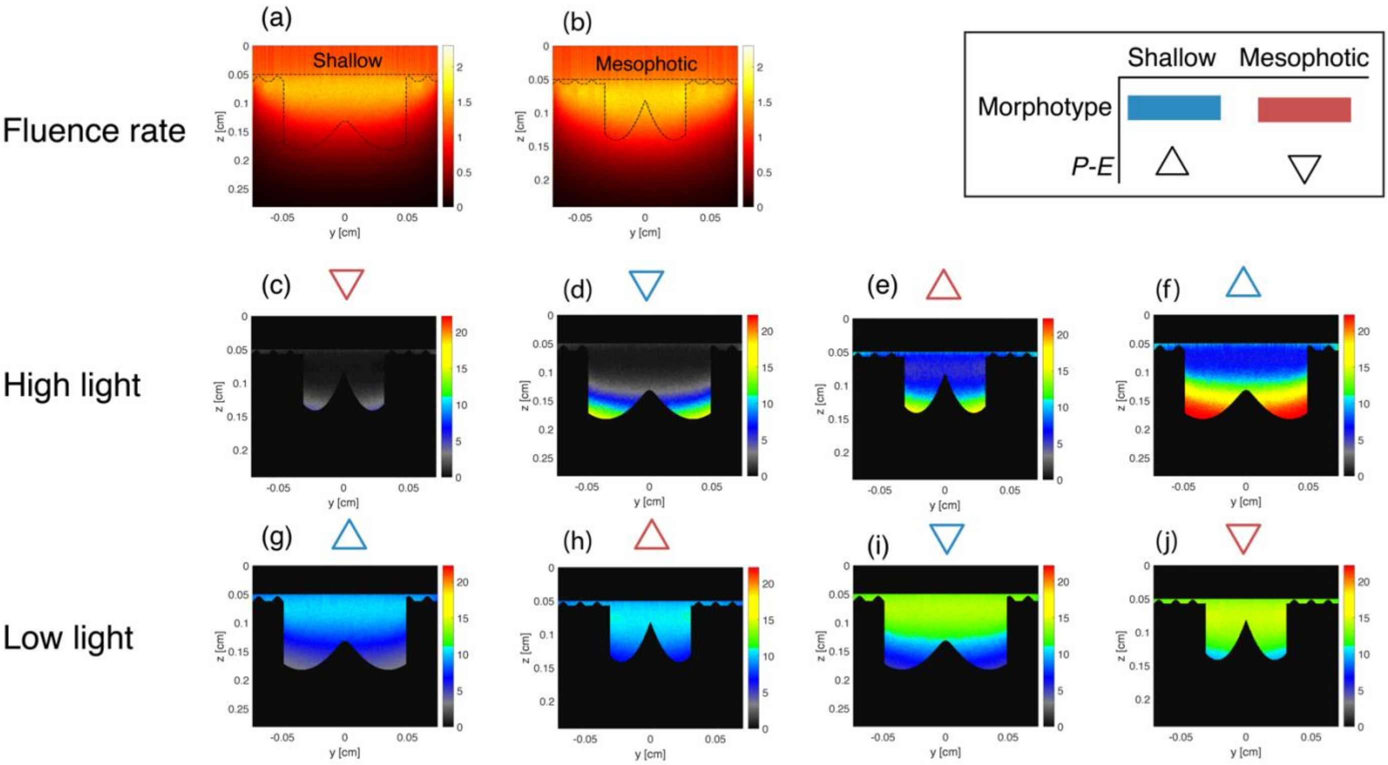

Figure S12. "No septae" scenario. Light propagation simulations shown in 2D (y-z axes) under light intensities of (**c-f**) 750 (high light) and (**g-j**) 45 (low light) µmol photons m^{-2} s^{-1}. **(a-b)** Relative fluence rates (delivered as W m^{-2}; "fire" color gradient) with contour indicating the surface boundaries. **(c-j)** Photosynthetic score ("rainbow" color gradient) on the tissue layers. Triangle color above the figures denotes the morphotype (shallow: *blue*; mesophotic: *red*), and the ambient photosynthetic performance (*P-E*) is represented by shape (shallow: triangle point up; mesophotic: triangle point down).

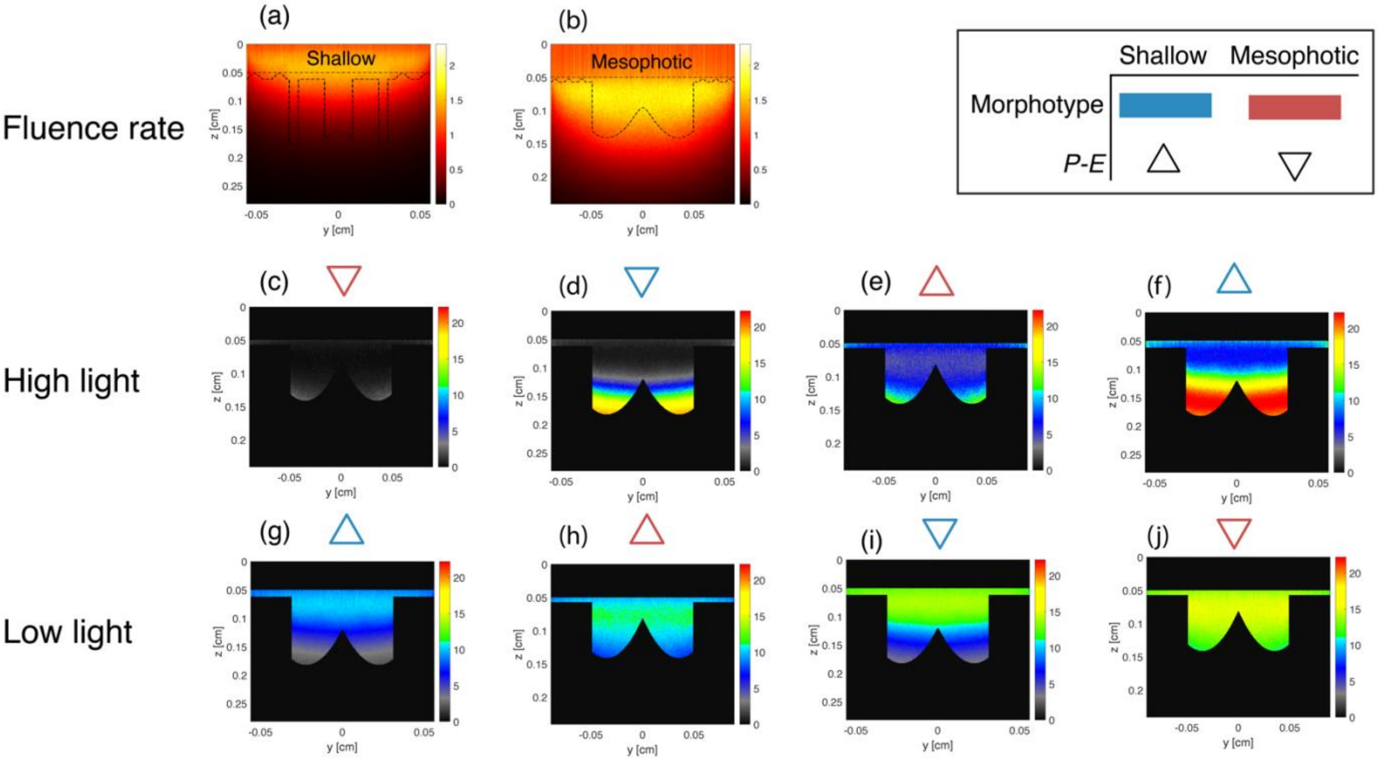

Figure S13. "Swap calyx diameter" scenario. Light propagation simulations shown in 2D (y-z axes) under light intensities of (**c-f**) 750 (high light) and (**g-j**) 45 (low light) µmol photons m^{-2} s^{-1}. **(a-b)** Relative fluence rates (delivered as W m^{-2}; "fire" color gradient) with contour indicating the surface boundaries. **(c-j)** Photosynthetic score ("rainbow" color gradient) on the tissue layers. Triangle color above the figures denotes the morphotype (shallow: *blue*; mesophotic: *red*), and the ambient photosynthetic performance (*P-E*) is represented by shape (shallow: triangle point up; mesophotic: triangle point down).

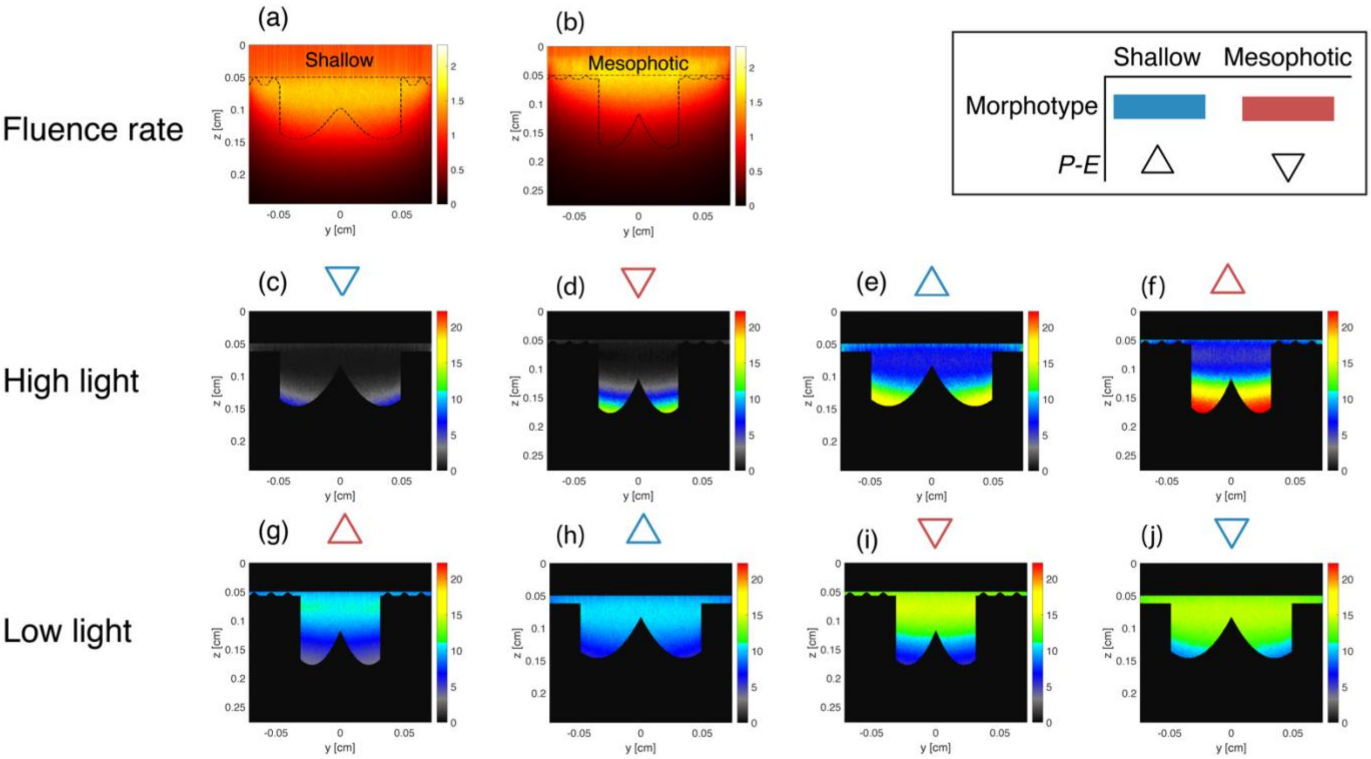

Figure S14. “Swap calyx height” scenario. Light propagation simulations shown in 2D (y-z axes) under light intensities of (**c-f**) 750 (high light) and (**g-j**) 45 (low light) µmol photons m^{-2} s^{-1}. **(a-b)** Relative fluence rates (delivered as W m^{-2}; “fire” color gradient) with contour indicating the surface boundaries. **(c-j)** Photosynthetic score (“rainbow” color gradient) on the tissue layers. Triangle color above the figures denotes the morphotype (shallow: *blue*; mesophotic: *red*), and the ambient photosynthetic performance (*P-E*) is represented by shape (shallow: triangle point up; mesophotic: triangle point down).

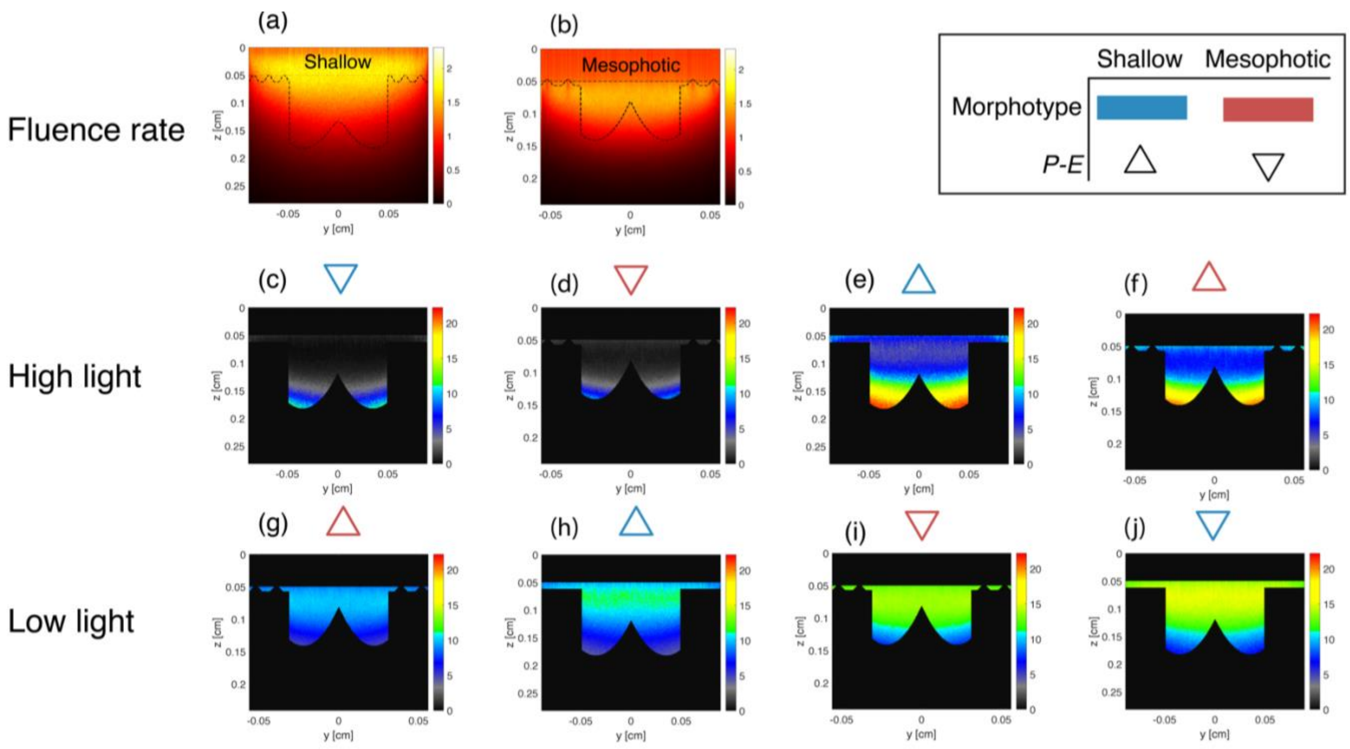

Figure S15. "Swap corallite spacing" scenario. Light propagation simulations shown in 2D (y-z axes) under light intensities of (**c-f**) 750 (high light) and (**g-j**) 45 (low light) µmol photons m^{-2} s^{-1}. **(a-b)** Relative fluence rates (delivered as W m^{-2}; "fire" color gradient) with contour indicating the surface boundaries. **(c-j)** Photosynthetic score ("rainbow" color gradient) on the tissue layers. Triangle color above the figures denotes the morphotype (shallow: *blue*; mesophotic: *red*), and the ambient photosynthetic performance (*P-E*) is represented by shape (shallow: triangle point up; mesophotic: triangle point down).

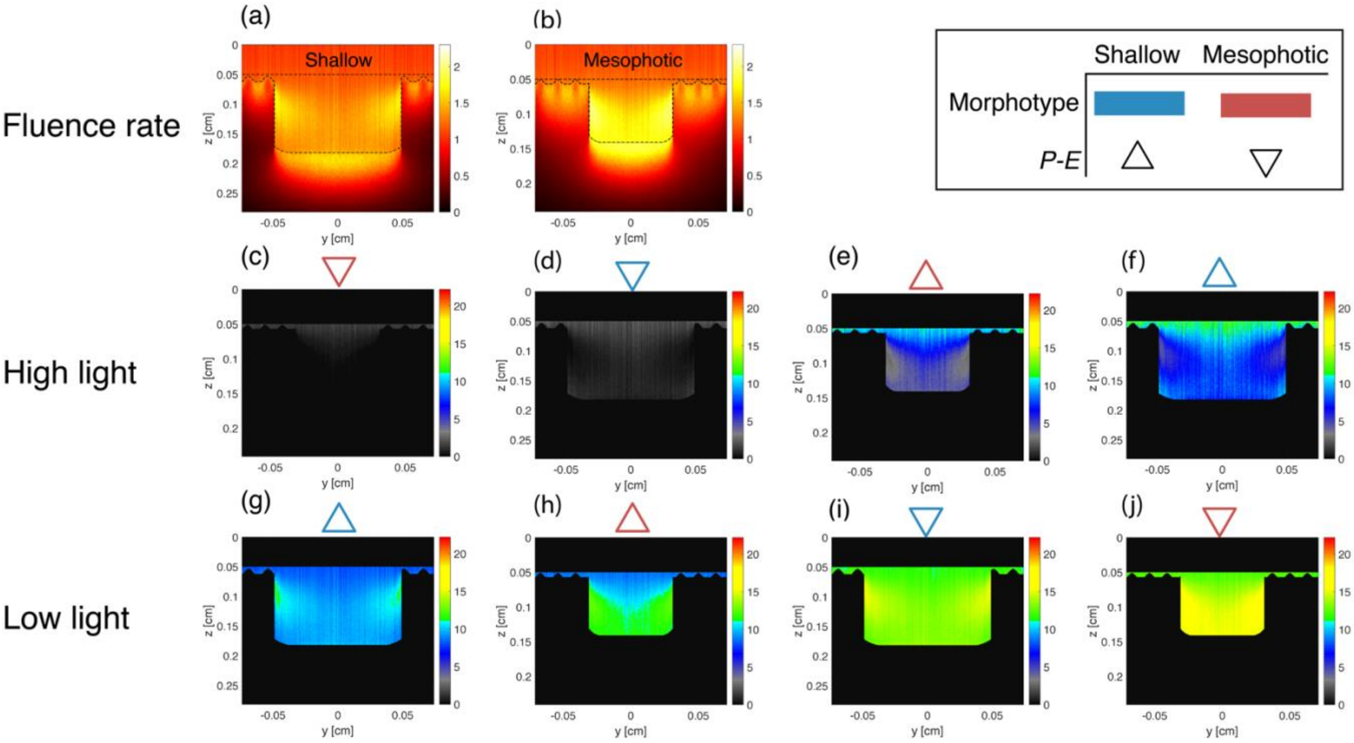

Figure S16. “Reduced tissue scattering without columella” scenario. Light propagation simulations shown in 2D (y-z axes) under light intensities of (**c-f**) 750 (high light) and (**g-j**) 45 (low light) µmol photons m^{-2} s^{-1}. **(a-b)** Relative fluence rates (delivered as W m^{-2}; “fire” color gradient) with contour indicating the surface boundaries. **(c-j)** Photosynthetic score (“rainbow” color gradient) on the tissue layers. Triangle color above the figures denotes the morphotype (shallow: *blue*; mesophotic: *red*), and the ambient photosynthetic performance (*P-E*) is represented by shape (shallow: triangle point up; mesophotic: triangle point down).

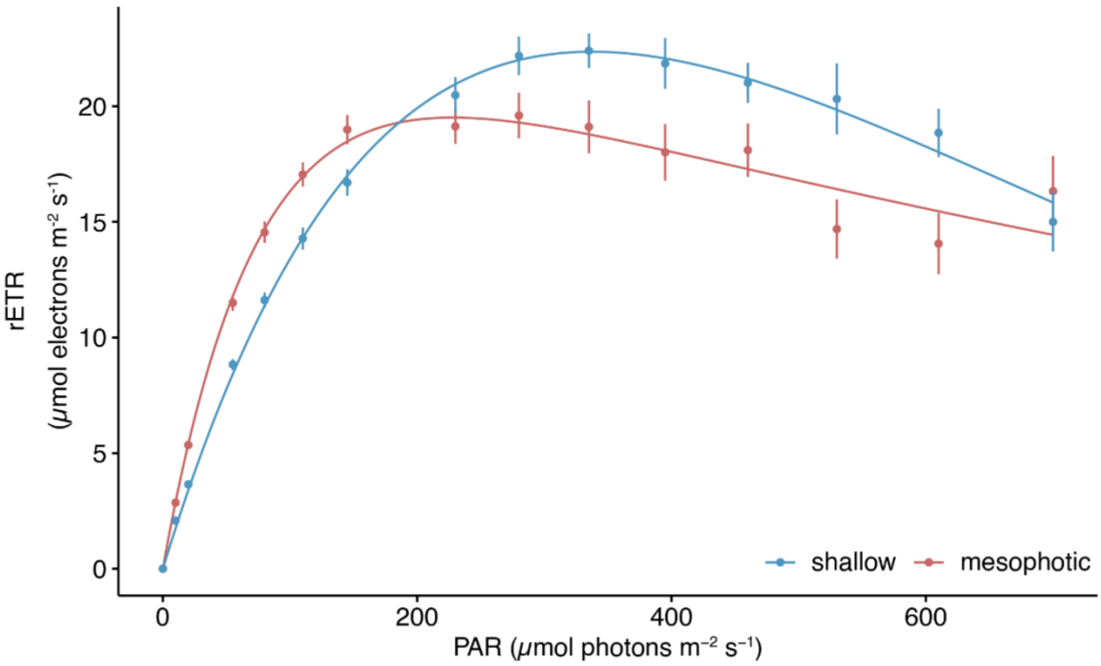

Figure S17. Relative electron transport rate (rETR) versus irradiance (photosynthetically active radiation, PAR, 400−700 nm) curves for shallow (*blue*) and mesophotic (*red*) corals (*n* = 6). Error bars are SE.
