# Supplementary material for: Morpho-functional traits of the coral Stylophora pistillata enhance light capture for photosynthesis at mesophotic depths
Source: Commun Biol. 2022 Aug 24;5:861. doi: 10.1038/s42003-022-03829-4 (PMC9402581; doi:10.1038/s42003-022-03829-4)
Supplement: Supplementary file 2 — Reporting Summary [file 42003_2022_3829_MOESM2_ESM.pdf]

nature portfolio

Corresponding author(s): Netanel Kramer

Last updated by author(s): Jul 7, 2022

Reporting Summary

Nature Portfolio wishes to improve the reproducibility of the work that we publish. This form provides structure for consistency and transparency in reporting. For further information on Nature Portfolio policies, see our Editorial Policies and the Editorial Policy Checklist.

Statistics

For all statistical analyses, confirm that the following items are present in the figure legend, table legend, main text, or Methods section.

n/a	Confirmed	
☐	☒	The exact sample size (n) for each experimental group/condition, given as a discrete number and unit of measurement
☐	☒	A statement on whether measurements were taken from distinct samples or whether the same sample was measured repeatedly
☐	☒	The statistical test(s) used AND whether they are one- or two-sided<br>*Only common tests should be described solely by name; describe more complex techniques in the Methods section.*
☐	☒	A description of all covariates tested
☐	☒	A description of any assumptions or corrections, such as tests of normality and adjustment for multiple comparisons
☐	☒	A full description of the statistical parameters including central tendency (e.g. means) or other basic estimates (e.g. regression coefficient) AND variation (e.g. standard deviation) or associated estimates of uncertainty (e.g. confidence intervals)
☐	☒	For null hypothesis testing, the test statistic (e.g. F, t, r) with confidence intervals, effect sizes, degrees of freedom and P value noted<br>*Give P values as exact values whenever suitable.*
☒	☐	For Bayesian analysis, information on the choice of priors and Markov chain Monte Carlo settings
☒	☐	For hierarchical and complex designs, identification of the appropriate level for tests and full reporting of outcomes
☒	☐	Estimates of effect sizes (e.g. Cohen's d, Pearson's r), indicating how they were calculated

Our web collection on statistics for biologists contains articles on many of the points above.

Software and code

Policy information about availability of computer code

Data collection	MATLAB (The MathWorks, Inc., 2022)
Data analysis	Statistical analyses were performed using the R software (v 4.1.3) and Dragonfly (© 2021 Object Research System (ORS) Inc.)

For manuscripts utilizing custom algorithms or software that are central to the research but not yet described in published literature, software must be made available to editors and reviewers. We strongly encourage code deposition in a community repository (e.g. GitHub). See the Nature Portfolio guidelines for submitting code & software for further information.

Data

Policy information about availability of data

All manuscripts must include a data availability statement. This statement should provide the following information, where applicable:

- Accession codes, unique identifiers, or web links for publicly available datasets
- A description of any restrictions on data availability
- For clinical datasets or third party data, please ensure that the statement adheres to our policy

All the code scripts, data, and other supplemental materials used for this study are available in the Dryad digital repository: https://doi.org/10.5061/dryad.7d7wm37w7

Field-specific reporting

Please select the one below that is the best fit for your research. If you are not sure, read the appropriate sections before making your selection.

☐ Life sciences ☐ Behavioural & social sciences ☒ Ecological, evolutionary & environmental sciences

For a reference copy of the document with all sections, see nature.com/documents/nr-reporting-summary-flat.pdf

Ecological, evolutionary & environmental sciences study design

All studies must disclose on these points even when the disclosure is negative.

Study description	We used 3D light models to describe the light propagation in small-scale skeletal features of corals from shallow and mesophotic Depths. Morphological variations between depths were tested using a mixed-effects permutational analysis (MEPA; 999 permutations) and included the sample ID as a random effect. Sample size was 30 corals.
Research sample	A total of 30 Stylophora pistillata corals were sampled due to permit restrictions and the long scan times associated with obtaining high-resolution 3D images
Sampling strategy	A total of 30 Stylophora pistillata corals were sampled due to permit restrictions and the long scan times associated with obtaining high-resolution 3D images
Data collection	Coral samples were collected during recreational and closed-circuit rebreather dives from shallow (4-5 m) and upper mesophotic (45-50 m) depths. After bleaching, the corals were scanned in a micro-CT and morphological analyses were performed using the Dragonfly software.
Timing and spatial scale	Corals were sampled in two dives during February 2021
Data exclusions	No data were excluded from the analyses
Reproducibility	All attempts to repeat the experiment were successful.
Randomization	The corals were allocated by depth of origin
Blinding	Full blinding was used during data acquisition and analysis

Did the study involve field work? ☒ Yes ☐ No

Field work, collection and transport

Field conditions	n/a
Location	The northern Gulf of Eilat, 29°30'05.3"N 34°55'01.9"E, at depths of 5 and 50 m
Access & import/export	Corals were collected under the Israel Nature and Parks Authority permit number 2020/42649
Disturbance	No disturbance was caused by the study

Reporting for specific materials, systems and methods

We require information from authors about some types of materials, experimental systems and methods used in many studies. Here, indicate whether each material, system or method listed is relevant to your study. If you are not sure if a list item applies to your research, read the appropriate section before selecting a response.

Materials & experimental systems

n/a	Involved in the study	
☒	☐	Antibodies
☒	☐	Eukaryotic cell lines
☒	☐	Palaeontology and archaeology
☐	☒	Animals and other organisms
☒	☐	Human research participants
☒	☐	Clinical data
☒	☐	Dual use research of concern

Methods

n/a	Involved in the study	
☒	☐	ChIP-seq
☒	☐	Flow cytometry
☒	☐	MRI-based neuroimaging

Animals and other organisms

Policy information about studies involving animals; ARRIVE guidelines recommended for reporting animal research

Laboratory animals	The study did not involve laboratory animals
Wild animals	Stylophora pistillata corals were sampled from 5 and 50 m depths and were bleached for morphometric analyses
Field-collected samples	Corals collected from the field were not maintained under laboratory conditions
Ethics oversight	Corals were collected under the Israel Nature and Parks Authority permit number 2020/42649

Note that full information on the approval of the study protocol must also be provided in the manuscript.
